# Supplementary material for: Rationale and protocol for a safety, tolerability and feasibility randomized, parallel arm, double-blind, placebo-controlled, pilot study of a novel ketone ester targeting frailty via immunometabolic geroscience mechanisms
Source: PLoS One. 2024 Sep 18;19(9):e0307951. doi: 10.1371/journal.pone.0307951 (PMC11410252; doi:10.1371/journal.pone.0307951)
Supplement: S1 File — (DOCX) [file pone.0307951.s001.docx]

**A randomized, double-blind, placebo-controlled, parallel group, feasibility pilot study to evaluate the tolerability and safety of a novel ketone ester ingredient in healthy older men and women.**

**Short Title:** Buck Institute Ketone Ester - RCT

**Study Code:** BIKE

**Protocol Number:** BUCK_2201

**PI:** Dr John Newman. MD PhD [jnewman@buckinstitute.org](mailto:jnewman@buckinstitute.org)

**Sponsor:** Buck Institute for Research on Aging

8001 Redwood Blvd, Novato, CA 94945

**Table of Contents**

[1. List of Abbreviations 5](#_Toc123899866)

[2. Protocol Synopsis 6](#_Toc123899867)

[3. Background/Rationale 7](#_Toc123899868)

[4. Objective 9](#_Toc123899869)

[5. Subjects 9](#_Toc123899870)

[5.1 Study Sample 10](#_Toc123899871)

[5.1.1 Inclusion Criteria 10](#_Toc123899872)

[5.1.2 Exclusion Criteria 10](#_Toc123899873)

[5.1.3 Excluded Medications/Supplements/Products 11](#_Toc123899874)

[5.2 Randomization 12](#_Toc123899875)

[6. Study Beverage 12](#_Toc123899876)

[7. Study Design and Visit Procedures 13](#_Toc123899877)

[7.1 Study Design 13](#_Toc123899878)

[7.2 Study Visit Procedures 13](#_Toc123899879)

[7.2.1 Recruitment 13](#_Toc123899880)

[7.2.2 Telephone Screen 13](#_Toc123899881)

[7.2.3 Visit 1: Screening (3 - 8 weeks before Baseline (Visit 3)) 13](#_Toc123899882)

[7.2.4 Visit 2: Ketone Kinetics Visit (at least 2 weeks after screening visit, at least 1 week before Baseline (Visit 3)) 14](#_Toc123899883)

[7.2.5 Visit 3: Baseline (Day 0) 15](#_Toc123899884)

[7.2.6 Daily at-home procedures (Day 1 – 14) 16](#_Toc123899885)

[7.2.7 Phone Check-In (Week 2, 6, 8 and 10) 16](#_Toc123899886)

[7.2.8 Daily at-home procedures (Day 15 - 84) 16](#_Toc123899887)

[7.2.9 Visit 4: Interim Visit (Week 4 – between Days 28 - 35) 17](#_Toc123899888)

[7.2.10 Visit 5: Final Visit (Week 12 – between Days 84 - 91) 17](#_Toc123899889)

[7.3 Study Schedule Summary 19](#_Toc123899890)

[7.4 Procedures by Clinic Visit 20](#_Toc123899891)

[7.4.1 Screening (Visit 1; at least 8 weeks before Visit 3, between Day -64 to -21) 20](#_Toc123899892)

[7.4.2 Kinetics Visit (Visit 2; at least 2 weeks after Visit 1, at least 1 week before Visit 3, between Day -7 to -42) 21](#_Toc123899893)

[7.4.3 Baseline (Visit 3; Day 0) 21](#_Toc123899894)

[7.4.4 Interim Safety Visit (Visit 4; between Day 28 - 35) 22](#_Toc123899895)

[7.4.5 Phone Check In (Weeks 2,6, 8 and 10) 22](#_Toc123899896)

[7.4.6 Final Study Visit (Visit 5; between Day 84 – 91) 23](#_Toc123899897)

[7.5 Study Beverages 23](#_Toc123899898)

[7.5.1 Description 23](#_Toc123899899)

[7.5.2 Study Beverage Nutritional Facts 24](#_Toc123899900)

[7.5.3 Labeling and Packaging 25](#_Toc123899901)

[7.5.4 Storage and Dispensing 25](#_Toc123899902)

[7.5.5 Blinding 25](#_Toc123899903)

[7.5.6 Access to Randomization Code 26](#_Toc123899904)

[7.6 Standard Breakfast – Kinetics Visit 26](#_Toc123899905)

[7.7 Clinical Measurements 28](#_Toc123899906)

[7.7.1 Physical characteristics, health updates and vital signs 28](#_Toc123899907)

[7.7.2 Laboratory Measurements 28](#_Toc123899908)

[7.7.3 Study Instructions/Reminder/Query 29](#_Toc123899909)

[7.7.4 Diet Recall 29](#_Toc123899910)

[7.7.5 Study Log and Beverage Tolerability Questionnaire 31](#_Toc123899911)

[7.7.6 Frailty Assessment 33](#_Toc123899912)

[7.7.7 Quality of Life Questionnaires 33](#_Toc123899913)

[7.7.8 Cognitive Testing 33](#_Toc123899914)

[7.7.9 Physical Testing 34](#_Toc123899915)

[7.7.10 Study Beverage/Standard Breakfast Administration 36](#_Toc123899916)

[7.7.11 Capillary Testing for Ketones and Glucose 36](#_Toc123899917)

[7.7.13 Continuous Glucose and Ketone sensor 37](#_Toc123899918)

[7.7.14 Stool Sample Collection 37](#_Toc123899919)

[8. Data Analysis and Statistical Methods 38](#_Toc123899920)

[8.1 Primary Outcome Variables 38](#_Toc123899921)

[8.2 Secondary Outcome Variables 38](#_Toc123899922)

[8.3 Exploratory Outcome Variables 38](#_Toc123899923)

[8.4 Sample Size 38](#_Toc123899924)

[8.5 Statistical Analysis 38](#_Toc123899925)

[8.5.1 Outcome Analysis 39](#_Toc123899926)

[8.5.2 Safety Analysis 40](#_Toc123899927)

[8.5.3 Missing or Incomplete Data 40](#_Toc123899928)

[9. Early Termination Procedures 40](#_Toc123899929)

[10. COVID-19 Management Procedures 41](#_Toc123899930)

[10.1 Recruitment: 41](#_Toc123899931)

[10.2 In-Clinic Study Visits: 41](#_Toc123899932)

[10.3 COVID-19 positive cases: 42](#_Toc123899933)

[11. Study Monitoring 42](#_Toc123899934)

[11.1 Concomitant Medication/Supplements and Treatment 42](#_Toc123899935)

[11.2 Compliance Monitoring 42](#_Toc123899936)

[11.3 Adverse Event Monitoring 43](#_Toc123899937)

[11.3.1 Grading and Severity 43](#_Toc123899938)

[11.3.2 Relationship 43](#_Toc123899939)

[11.3.3 Serious Adverse Event Definition/Qualification 44](#_Toc123899940)

[11.3.4 Serious Adverse Event Reporting Instructions 44](#_Toc123899941)

[11.3.5 CRF Recording of Adverse Events 45](#_Toc123899942)

[11.3.6 Serious Adverse Event Follow-Up 45](#_Toc123899943)

[11.3.7 Pregnancy 45](#_Toc123899944)

[12. Conduct of the Study 46](#_Toc123899945)

[12.1 Ethics and Regulatory Considerations 46](#_Toc123899946)

[12.2 Institutional Review Board 46](#_Toc123899947)

[12.3 Informed Consent 46](#_Toc123899948)

[12.4 Subject Confidentiality 47](#_Toc123899949)

[12.5 Withdrawal of Subjects from the Study 47](#_Toc123899950)

[12.6 Incidental Findings 47](#_Toc123899951)

[12.7 Changes to the Protocol 47](#_Toc123899952)

[12.8 Protocol Deviations and Violations 48](#_Toc123899953)

[12.9 Case Report Forms 48](#_Toc123899954)

[12.10 Monitoring 48](#_Toc123899955)

[12.11 Auditing 49](#_Toc123899956)

[12.12 Records and Sample Retention 49](#_Toc123899957)

[12.13 Termination of Study 49](#_Toc123899958)

[12.14 Disclosure 49](#_Toc123899959)

[12.15 References 50](#_Toc123899960)

# List of Abbreviations

| **ADL** | activities of daily living |
| --- | --- |
| **AE** | adverse event |
| **BHB** | beta-hydroxybutyrate |
| **BMI** | body mass index |
| **BTQ** | beverage tolerability questionnaire |
| **CFR** | Code of Federal Regulations |
| **CRF** | case report form |
| **FDA** | Food and Drug Administration |
| **FDR** | false discovery rate |
| **g** | gram |
| **h** | hour(s) |
| **GCP** | good clinical practice |
| **GRAS** | generally recognized as safe |
| **HIPAA** | Health Insurance Portability and Accountability Act |
| **IADL** | instrumental activities of daily living |
| **ITT** | intent-to-treat |
| **ICH** | International Conference on Harmonization |
| **IRB** | Institutional Review Board |
| **KE** | ketone esters |
| **kg** | kilogram |
| **LDL** | low-density lipoprotein |
| **m^2^** | meter squared |
| **MCT** | medium-chain triglycerides |
| **mg** | milligram |
| **min** | minute |
| **mL** | milliliter |
| **mM** | millimolar |
| **mm Hg** | millimeters of mercury |
| **PP** | per protocol |
| **RANOVA** | repeated measures analysis of variance |
| **RANCOVA** | repeated measures analysis of covariance |
| **RPE** | rating of perceived exertion |
| **SAE** | Serious Adverse Event |
| **SOP** | standard operating procedure |
| **TSH** | thyroid stimulating hormone |
| **y** | year |
|  |  |

# Protocol Synopsis

| **Study Title** | A randomized, double-blind, placebo-controlled, parallel study to evaluate the tolerability and safety of a novel ketone ester ingredient in healthy older men and women. |
| --- | --- |
| **Short title** | Buck Institute Ketone Ester – RCT (**BIKE**) |
| **Sponsor** | Buck Institute for Research on Aging, Novato, CA, USA |
| **Site** | Buck Institute for Research on Aging, 8001 Redwood Blvd, Novato, CA 94945, USA |
| **Investigator** | John Newman, MD, PhD |
| **Study Design** | Double-blind, randomized, placebo-controlled, parallel study |
| **Study Subjects** | Generally healthy adults at or over the age of 65 years old |
| **Planned Sample Size** | 30 subjects (15 per group) |
| **Study Beverages** | Tropical flavored beverages containing:  Ketone ester (KE) (Chemical name: Bis-octanoyl-(R)-1,3-butanediol, Common name: C8 ketone di-ester)  OR  Placebo (non-ketogenic canola oil)  *Optional second acute ketone kinetics visit*  *Unflavored ketone power reconstituted in water containing:*  *Ketone ester (KE) (Chemical name: Bis-octanoyl-(R)-1,3-butanediol, Common name: C8 ketone di-ester)* |
| **Serving Size** | KE:12.5 g/day for 1 week, 25 g/day for 11 weeks  Placebo (non-ketogenic canola oil) 12.5 g/day for 1 week, 25 g/day for 11 weeks |
| **Planned Study Period** | 15 - 20 weeks involvement per subject, depending on time between screening and baseline visit.  18 months from the first subject starting to the last subject complete.  *Optional second acute ketone kinetics visit after the end of the main trial.* |
| **Outcomes** | |
| **Primary** | The primary outcome measure is the proportion of subjects reporting the same moderate to severe symptom (among dizziness, headache or nausea) occurring on more than one day within any given recall period (after week 0 - 2 acclimation period) when ketone esters are consumed daily for 12 weeks. |
| **Secondary** | Safety: study beverage-emergent adverse events reported by subjects, vital signs (blood pressure, heart rate, body temperature), body weight, and changes in clinical laboratory measurements (chemistry panel [including thyroid hormones], hematology panel, lipid panel [including apolipoprotein B]) when ketone esters are consumed daily for 12 weeks.  Describe the short-term blood ketone and glucose changes in adults ≥65 y after consuming a single serving of either 12.5 g or 25 g of ketone ester beverage. |
| **Exploratory** | Differences in physical function between groups at the end of the study  Differences in cognitive function between groups at the end of the study  Differences in quality-of-life scores between groups at the end of the study  Differences in the gut microbiome between groups over the course of the study  Difference in biomarkers of aging between groups over the course of the study  Describe the short-term blood ketone and glucose changes in adults ≥65 y after consuming a single serving of either 12.5 g or 25 g of ketone ester powder reconstituted in water. |

# Background/Rationale

Beta-hydroxybutyrate (BHB) is one of the three physiological ‘ketone bodies’ produced endogenously from fatty acids in the setting of a ketogenic diet, fasting/starvation, or following strenuous exercise. In modern humans, consuming a mixed diet containing a significant carbohydrate content, BHB levels are typically low (< 0.1 mM). Under ketogenic conditions BHB levels in the blood increase and a natural state of nutritional ketosis develops (BHB ≥ 0.5 mM). BHB functions as a metabolic fuel to facilitate adenosine triphosphate generation,^1^ particularly in the brain, heart and skeletal muscle. Additionally, growing evidence suggests that BHB can act as a signaling metabolite, ultimately impacting overall health.^2, 3^

An alternative method to elevate blood ketone levels is to consume exogenous ketones. Exogenous ketones contain ketones or ketone precursors that break down into ketone, to elevate blood BHB concentrations without the need for natural ketone production. Whilst the BHB delivered is from an external source, it is oxidized by the same pathway as endogenously produced BHB.^4^ Published data suggests that exogenous ketone supplements might have positive impacts on health and performance of people as they age. For example, human studies of exogenous ketones have found beneficial effects on endurance exercise performance,^5^ cognitive performance,^6^ maintaining healthy blood glucose,^7^ supporting immune responses ^8^ and supporting healthy cardiac function.^9^ Animal studies are more advanced, with data demonstrating efficacy in a range of health-related conditions such as supporting healthy cognitive function,^10, 11^ body weight control,^12^ and encouraging a healthy immune response.^13^

**Figure 1**: Chemical structure of ketone esters bis hexanoyl R-1,3-butanediol (C6 ketone diesterBH-BD) and bis-octanoyl R-1,3-butanediol (C8 ketone di-ester, BO-BD) and their metabolism to beta hydroxybutyrate (BHB) and acetoacetate (AcAc).


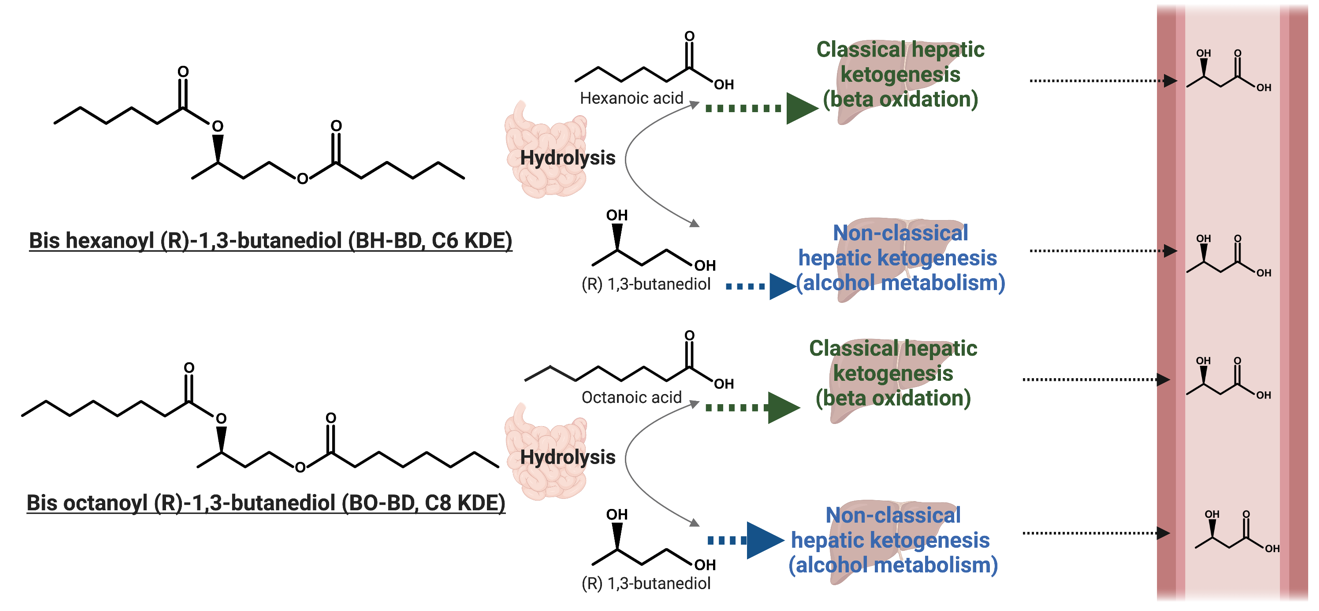


A range of commercially available exogenous ketones have emerged in recent years. Of these products, ketone esters (KE) are considered to be a preferred form as they deliver no free acid or mineral load and generally have high bioavailability compared to other forms, such as ketone salts.^14^ Ester compounds are regularly consumed as part of the diet and ester bonds are hydrolyzed by ubiquitously expressed and non-specific esterase enzymes in the gut and in the plasma. Once the ester bonds in KE are hydrolyzed, the hydrolysis products are metabolically converted into ketone bodies (**Figure 1)**. Three KE have been described in the scientific literature to date, firstly the BHB-monoester which contains BHB esterified to the ketogenic precursor (R)-1,3-butanediol,^15^secondly the C6 Ketone di-ester which consists of two molecules of ketogenic fatty acid (hexanoic acid) esterified to (R)-1,3-butanediol,^16^ and finally a di-ester of two molecules of acetoacetate esterified to (R,S)-1,3-butanediol.^17^All of these KE are “Generally Recognized as Safe” (GRAS) and are commercialized as an ingredient in health, sports performance and recovery beverages containing 5 - 30 g of KE (KetoneAid, USA; TdeltaS Global, USA; BHB Therapeutics Ltd // Juvenescence, USA) or in softgel capsules (KetoLogic, USA). Recently, efforts to improve the sensory properties of the C6 Ketone Di-ester resulted in the development of a di-ester of two molecules of the ketogenic fatty acid (octanoic) acid and (R-1,3-butanediol), C8 Ketone di-ester. The C8 ketone di-ester has superior palatability to the C6 ketone di-ester whilst having similar metabolic fate^18^ (Figure 1), safety and functional properties to this molecule as well as to other ketone-based FDA notified GRAS substances (GRN 515, GRN 1032) (See Safety Assessment, Kruger 2022). C8 ketone di-ester obtained a GRAS conclusion in July 2023, based on its’ similarity to C6 ketone di-ester and commercial products containing C8 ketone di-ester are now available.

KE have undergone comprehensive preclinical safety testing ^19, 20^ as well as extensive studies in young adults, confirming their safety and tolerability. However, their tolerance in an older population is unknown. For example, the GRAS assessment of the C6 ketone di-ester included a subchronic toxicity evaluation in rodents, and safety and study of the KE in healthy adults between the age of 18 - 65 years old.^16^ Subjects consumed up to 25 g per day of KE in a 75 mL specialty beverage or a placebo. No clinically meaningful changes in safety measures including vital signs and clinical laboratory measurements were detected within or between groups. There were no differences in at-home composite systemic and gastrointestinal tolerability scores between KE and placebo at any time in the study, or in acute tolerability measured 1-hour post-consumption in-clinic. Similarly, several multi-day studies of the BHB monoester found that up to 75 g daily was well tolerated with few side-effects, ^15, 21, 22^ although those side effects did include vomiting, abdominal pain, nausea, flatulence, heartburn, headache, dizziness ^6, 15, 23-25^ some of which are similar to those commonly seen during adaptation to the ketogenic diet. Whilst we expect that KEs such as the BHB monoester, C6 ketone di-ester and C8 ketone di-ester will have broadly similar safety, tolerability and metabolic effects in younger and older adults, it is unknown whether age-related changes in ketogenic gene regulation, ^26^or in hepatic or renal function could impact KE metabolism or tolerability. Therefore, the primary goal of this project is to rigorously test the tolerability and safety of KE in older adults when taken daily for 12 weeks.

This randomized, double-blind, placebo-controlled trial aims to characterize the tolerability and safety of daily consumption of the C8 ketone di-ester in healthy older adults over 12 weeks. Subjects will be asked to complete Beverage Tolerability Questionnaires (BTQ) to rate any side effects occurring while they are taking the KE. Safety will also be assessed by collection of blood and urine samples, vital signs, body weight, and monitoring of adverse events (AEs). In order to determine the acute changes in blood ketones and glucose after two serving sizes of ketone ester, subjects will complete a single ‘kinetics visit’ where serial capillary blood samples will be collected after ingestion of one study beverage. As a new form of the ketone ester product (unflavored powder that is reconstituted into a beverage) recently became available, subjects may complete an optional second acute ketone kinetics visit at the end of the main study that will compare the subjects’ ketone responses to the reconstituted powder to the data collected following the beverage. The study will also collect pilot data to explore possible effects of KE on physical function, cognitive function and quality of life. The findings of this study will be used to facilitate future mechanistic studies of KE in aging.

# Objective

The primary objective of this study is to investigate the tolerability of a novel ketone ester taken daily for 12 weeks in healthy men and women at, or over the age of 65.

The secondary objectives are to investigate the safety of 12 weeks of ketone ester consumption in this population and to characterize the acute changes in blood ketone and glucose after two serving sizes of ketone ester beverage.

Additional exploratory outcomes will investigate any physical, cognitive or quality of life changes occurring with ketone ester consumption, and to characterize the acute changes in blood ketones and glucose after two serving sizes of ketone ester powder reconstituted in water.

# Subjects

This investigation will recruit a group of 30 community-dwelling, activities of daily living (ADL) -independent older adults at or over the age of 65 years old. We will recruit roughly equal portions of men and women.

## Study Sample

Each subject must meet all of the following inclusion criteria and none of the exclusion criteria at baseline (Visit 3; Day 0) in order to participate in this study. This will be documented in the Case Report Form (CRF).

### Inclusion Criteria

1. Subject is greater than or equal to 65 years of age, inclusive at Visit 1.
2. Subject has a BMI 18.5-34.9 kg/m^2^ (inclusive) at Visit 1.
3. Subject is willing and able to comply with all study procedures including randomization into any of the experimental groups, maintenance of habitual dietary intake, exercise and medication and supplement use, blood draws and the following prior to test visits: fasting (≥10 h; water only), no alcohol (≥ 10 h), no cannabis products (≥10 h) and no exercise (≥ 10 h).
4. Subject has no health conditions that would prevent them from fulfilling the study requirements as judged by the Clinical Investigator on the basis of medical history and routine laboratory test results.
5. Subject understands the study procedures and signs forms providing informed consent to participate in the study.

### Exclusion Criteria

1. Subject is non ambulatory
2. Subject has a CSHA clinical frailty score > 5
3. Subject requires assistance with any activity of daily living, excluding continence
4. Subject lives in an institutional setting (skilled nursing facility or residential care facility for the elderly).
5. Subject is a female who has not passed menopause.
6. Subject is unable to converse in English
7. Subject is unable to provide informed consent due to cognitive impairment or insufficient English language comprehension
8. Subject has been hospitalized within 30 days of Visit 1, 2 or 3.
9. Subject has an abnormal laboratory test result(s) of clinical importance, indicating unstable chronic disease of major organ dysfunction, at Visit 1, at the discretion of the Medical Officer. One re-test will be allowed on a separate day prior to Visit 2, for subjects with abnormal laboratory test results.
10. Subject has a history or presence of uncontrolled and/or clinically active pulmonary, cardiac (e.g. >= New York Heart Association class III), hepatic, renal, endocrine (including type 1 diabetes), hematologic, immunologic, neurologic (e.g., Alzheimer’s or Parkinson’s diseases), psychiatric (including unstable depression and/or anxiety disorders) or biliary disorders. Stable chronic disease is not an exclusion criterion unless specified.
11. Subject has a clinically important gastrointestinal condition that would potentially interfere with the evaluation of the study beverage [e.g., inflammatory bowel disease, irritable bowel syndrome, chronic constipation, severe constipation (in the opinion of the Clinical Investigator), history of frequent diarrhea, history of surgery for weight loss, gastroparesis, systemic disease that might affect gut motility according to the Investigator, reflux requiring daily medication, history of gastrointestinal ulcers or bleeding, and/or clinically important lactose intolerance].
12. Subject has a history of alcohol or substance abuse.
13. Subject is consistently using prescriptive or over-the counter medications where alcohol is a contraindication at the discretion of the Investigator.
14. Subject has a known allergy, intolerance, or sensitivity to any of the ingredients in the study beverages, including soy and milk protein.
15. Subject has uncontrolled hypertension (systolic blood pressure ≥140 mm Hg or diastolic blood pressure ≥90 mm Hg) as defined by the blood pressure measured at Visit 1. One re-test will be allowed on a separate day before Visit 2, for subjects with abnormal blood pressure.
16. Subject is undergoing treatment or active surveillance for cancer, or has been diagnosed with cancer in the prior two years, except for non-melanoma skin cancer.
17. Subject has recently used antibiotics within 30 days of Visit 1, 2 or 3.
18. Subject has extreme dietary habits (e.g., intermittent fasting or time restricted eating, Atkins diet, vegan, very high protein/low carbohydrate or has used weight-loss medications (including over-the-counter medications and/or supplements) or programs within 30 days of Visit 1, 2 or 3.
19. Subject has used medications (over-the-counter or prescription) known to influence gastrointestinal function including, but not limited to, opioids, weight loss medications, antidiarrheals, and antispasmodics) within 30 days of Visit 1, 2 or 3.
20. Subject has used ketone supplements (ketone salts or esters, and medium chain triglycerides [MCT]) within 30 days of Visit 1, 2 or 3.
21. Subject has unstable use of thyroid, antihypertensive, antidepressant, or statin medications within 30 days of Visit 1, 2 or 3.
22. Subject has a condition the Clinical Investigator believes would interfere with their ability to provide informed consent, comply with the study protocol, which might confound the interpretation of the study results, or put the subject at undue risk.
23. Subject works nights or shifts that means it is not possible to maintain a consistent meal schedule during the study.
24. Subject is not permitted to visit the Buck Institute campus, for example due to inability to confirm COVID-19 vaccination status.
25. Subject does not have a Bluetooth enabled smartphone.
26. Subject does not have access to the internet

### Excluded Medications/Supplements/Products

Use of thyroid hormone therapy, statins, antihypertensives, antidepressants, constipation medications should be **stable** for the 30 days prior to Visit 1, 2 or 3. Additionally, use of any antibiotic therapy is not permitted within 30 days of Visit 1, 2 or 3 and throughout the study period. Subjects should not use opioids, weight loss medications, antidiarrheals, antispasmodics, ketone supplements (including MCT oil), or other medications (over-the-counter or prescription) or dietary supplements known to alter gastrointestinal function within 30 days of Visit 1, 2 or 3 and throughout the study period, with the exception of stable use of constipation medications and supplements.

Should a subject require any of these medications or supplements, the study staff should consult with the Investigator to discuss the subject’s continued participation in the trial. At the discretion of the Medical Officer in consultation with the Investigator, subjects may suspend consumption of the Study Product and completion of the Study Log for up to 10 days and re-start the protocol at the point of suspension.

## Randomization

If a subject meets all inclusion and none of the exclusion criteria, the following steps should occur at Visit 2 (Kinetics Visit):

1. A block randomization sequence (block size 4) will be prepared by a statistician. It will result in a 1:1:1:1 breakdown of subjects to receive either 12.5 g or 25 g of ketone ester at the kinetics visit followed by ketone ester or placebo beverage for the 12 week study. Possible combinations are 12.5g / KE, 12.5 g / Placebo, 25 g /KE, 25 g / Placebo.
2. If a subject meets all inclusion and none of the exclusion, a staff member will randomize the subject.
   1. Note: for the kinetics visit, the study team and subject will not be blinded to the ketone ester serving size allocation. The outcomes of interest: blood ketone and glucose concentrations, are not subject to conscious control and will not be influenced by knowledge of serving size allocation. The same serving size will be used at the optional second kinetics visit using the ketone ester powder.
   2. Note: for the 12 week study the study team and subject will be blinded to the study beverage allocation. See details of blinding below.
3. The randomization number will indicate the ketone ester serving size for the kinetics visit and the study beverage code allocation for the 12-week study..
4. The randomization number will be recorded in the subject's CRF.

# Study Beverage

Kinetics Visit (Visit 2)

- 12.5g of KE in a tropical flavored beverage
- 25 g of KE in a tropical flavored beverage

12-week study

- KE; 12.5 g/day for 1 week and 25 g/day for 11 weeks
- Placebo (non-ketogenic canola oil) 12.5 g/day for 1 week, 25 g/day for 11 weeks

Optional second kinetics visit

- 12.5g of KE as a unflavored powder reconstituted in water
- 25 g of KE as a unflavored powder reconstituted in water

Study products will be supplied by BHB Therapeutics Ltd (a subsidiary of Juvenescence).

For the 12-week study, subjects will be dispensed study beverages and instructed to consume half of a bottle per day in week 1, or one full bottle per day in weeks 2 through 11- to be consumed within 5 minutes after eating their first meal of the day. Further details follow below.

# Study Design and Visit Procedures

## Study Design


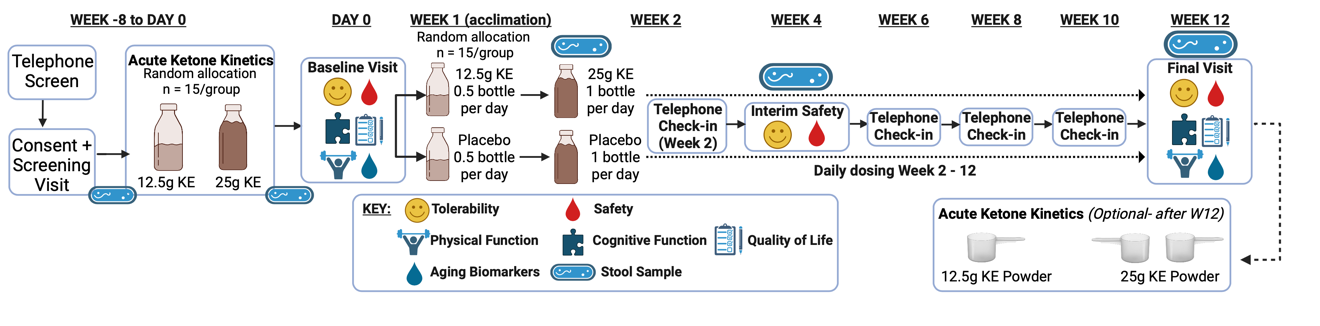


## Study Visit Procedures

### Recruitment

Recruitment will rely on sharing of a promotional flier (Appendix 1) physically and digitally via email and social media, along with targeted direct mail from existing mailing lists. Recruitment will occur from communities around Novato, CA and the greater northern San Francisco Bay Area. The Buck Institute has extensive community engagement programs including newsletters, tours, visiting speakers, and adult learning which have resulted in a contact list of 13,000 individuals estimated to be 70% over age 65. The Buck team will leverage existing relationships with senior service providers in Marin County, such as Vivalon, to help recruit subjects. Additional targeted advertising and community group engagement in the nearby communities of Santa Rosa, San Rafael, and Marin City will enhance outreach to black and/or Hispanic potential subjects.

### Telephone Screen

A waiver of consent will be obtained in order to collect subject data relating to activities of daily living and major health conditions during the telephone screen. The rationale for this, is to reduce subject burden by decreasing the number of subjects who attend an in-person screening visit but have easily identifiable functional deficits that make them ineligible to participate. All subjects will be allocated a screening number.

During the telephone screen subjects will be provided a brief summary of the study and assessed for initial eligibility. Eligibility screening will include asking about major exclusion criteria including institutional living, ambulation status, activities of daily living (ADL) dependence and recent hospitalizations. Following this screening call, eligible subjects will be provided with the Informed Consent Form for review ahead of the in-person Screening Visit. Phone Screen script can be found in Appendix 2. Subjects who may still be eligible after the telephone screen will be invited to schedule an in-person screening visit, and the Informed Consent Form will be shared via email for subject to review ahead of the visit.

### Visit 1: Screening (3 - 8 weeks before Baseline (Visit 3))

Subjects must meet pre-test requirements on arrival for Visit 1 (fasting ≥10h, no alcohol ≥10h, no exercise ≥10h, no cannabis products ≥10h); a reminder email will be sent out ahead of the visit. On arrival subjects will provide Informed Consent before any study-specific procedures are performed. Consent will be obtained by a suitably qualified team member who is trained to ensure appropriate understanding of the study via teach-back and other methods, and can answer any questions the subject may have pertaining to the study. Subjects will be asked if they consent to the optional Visit 6 (second acute ketone kinetics visit), and this will be recorded.

Following consent, demographic characteristics, height, weight, waist circumference, BMI, vital signs, last menses/menopause query (female only) and current medication/supplement use will be assessed, and recorded in the CRF. Inclusion/exclusion criteria will be reviewed in detail and recorded in the CRF, informed by a medical history interview. This will include an assessment of Katz’s Activities of Daily Living (ADL)^27^and Lawton’s Instrumental Activities of Daily Living (IADLs)^28^and CSHA Frailty Score (Canadian Study of Health and Aging).^29^  Fasting blood samples will be collected for the following analysis: clinical chemistry, hematology, lipid profile, thyroid hormones. A clean catch urine sample will be collected for urinalysis. Subjects will have the opportunity to taste both study beverages (5 – 15 mL) to evaluate palatability, and confirm subjects’ willingness to consume the beverage daily over the course of the study. Subjects will be given a stool sample collection kit (instructions insert shown in Appendix 3) to take home for at-home sample collection up to 3 days before Visit 2, the stool sample will be returned to investigators using a prepaid mailer. Finally, the study instructions will be reviewed and subjects will be given the chance to ask any questions about the study.

Following receipt of the laboratory test results, the study Medical Officers will review the subject intake information and confirm eligibility with the Investigator (this must take place within 2 weeks of screening visit). Subjects who meet all of the inclusion and none of the exclusion criteria will be randomized to receive one of two ketone ester serving sizes (12.5 g or 25 g) for the ketone kinetics visit (Visit 2) AND into one of the two experimental groups (ketone ester or placebo) (see Section 4.2 - Randomization) and allocated an enrollment number.

### Visit 2: Ketone Kinetics Visit (at least 2 weeks after screening visit, at least 1 week before Baseline (Visit 3))

Subjects must meet pre-test requirements on arrival for Visit 2 (fasting ≥10h, no alcohol ≥10h, no exercise ≥10h, no cannabis products ≥10h); a reminder email will be sent out ahead of the visit. Subjects will be queried on any changes in their health that may affect their inclusion in the study. If anything has changed between Visit 1 and Visit 2, subjects may delay Visit 2 for up to 30 days to allow them to meet the inclusion criteria.

Subjects will complete a baseline BTQ to screen for any baseline subjective symptoms, and a baseline BHB and glucose reading will be taken using capillary blood obtained from a fingerstick. Subjects will be given a standard breakfast meal. Within 5 minutes of finishing the meal, a study beverage will be administered (either half a bottle, containing 12.5 g of KE or a full bottle, containing 25 g of KE) and consumed within 5 minutes. Subjects may be asked to wear a nose clip, or asked to self-administer the beverage through a syringe into the back of the mouth to reduce their ability to taste the study beverage. A timer will be started directly after the subject finishes the study beverage. Fingerstick blood samples will be collected for BHB and glucose determination 30, 60, 90, 120, 180 and 240 minutes after study beverage consumption.

After the final fingerstick blood sample, subjects will complete a second BTQ and will receive a snack. Subjects will be shown and instructed on the use of the equipment for the physical function testing and complete a brief familiarization. Subjects will be given a wearable actigraphy device (Fitbit, USA) and instructed on its use during the study. Subjects will be given a stool sample collection kit (instructions insert shown in Appendix 3) to take home for at-home sample collection up to 3 days before Visit 3, the stool sample will be returned to investigators using a prepaid mailer. Subjects will be shown how to use the online diet recall tool and asked to complete this for one day prior to the baseline visit (Visit 3). They will be reminded of the pre-test instructions for the baseline visit. (Visit 3)

### Visit 3: Baseline (Day 0)

Subjects must meet pre-test requirements on arrival for Visit 3 (fasting ≥10h, no alcohol ≥10h, no exercise ≥10h, no cannabis products ≥10h); a reminder email will be sent out ahead of the visit. On arrival subjects will be queried to ensure compliance with study instructions, changes to the subject’s health and medication/supplement use will be assessed, and subject’s compliance with inclusion/exclusion criteria will be reviewed. If anything has changed between Visit 2 and Visit 3, subjects may delay Visit 3 for up to 30 days to allow them to meet the inclusion criteria, providing Visit 3 occurs within 8 weeks of Visit 1.

Fasting blood samples will be collected for the following analysis: clinical chemistry, hematology, lipid panel, liver function, thyroid function and mechanistic aging biomarker investigations (further details below). Additional samples will be retained to bank for possible future mechanistic investigations. Then, subjects will be asked to collect a clean catch urine sample (for urinalysis). Subjects will be given access to a selection of snacks after these samples are collected.

Then subjects will complete the following paper questionnaires: Profile of Mood States (Short form),^30^The Sexual Quality of Life Questionnaire (Male or Female Version),^31, 32^ Short Form Health Survey-36,^33^ Pittsburgh Sleep Quality Index,^36^ Pittsburgh Fatiguability Scale,^34^ Geriatric Depression Scale.^35^(Appendix 4).

After these questionnaires are complete, body weight, waist circumference and vital signs will be recorded.

Subjects will complete cognitive function testing: Montreal Cognitive Assessment,^36^Digit Symbol Substitution Task^37^ and Trails A and B^38^ (Appendix 4) Subjects will complete physical function testing: Short Physical Performance Battery,^39^ 1 rep max leg press,^40^ sub maximal leg press repetitions to failure, 6-minute walk test ^41^and grip strength.^42^

Subjects will be given a stool sample collection kit (instructions insert shown in Appendix 4) to take home for at-home sample collection 6 - 9 days after Visit 3, the stool sample will be returned to investigators using a prepaid mailer. If available, subjects will be shown how to apply a continuous glucose and ketone sensor (Abott Bio wearables, CA, USA) to their outer upper arm, and supervise self-application. Subjects will be instructed on how to sync the sensor and how to remove it after 14 days.

Subjects will be given sufficient study products for 4 weeks and the Study Log (Part I (Appendix 5). They will be asked to consume their first serving of study product with their next meal after the test visit. They will be reminded of study instructions (for study beverage consumption, Study Log completion and to maintain habitual exercise, meal/diet and medication/supplementation use).

### Daily at-home procedures (Day 1 – 14)

Each day at home, subjects should consume their first meal of the day at a similar time and consume the study product within 5 minutes of finishing their first meal. Subjects will complete the Study Log immediately before they consume their second meal of the day or a snack (3 - 6 h after the study beverage) to confirm they consumed their study product. The Study Log will query the presence of specific symptoms with a beverage tolerance questionnaire (BTQ). Between days 6 - 9, subjects will collect a stool sample using the provided kit. If worn, the continuous glucose and ketone sensor should be removed at home on day 14.

### Phone Check-In (Week 2, 6, 8 and 10)

Subjects will schedule a phone call with the Study Team in which they will verbally complete the BTQ for symptoms occurring in the preceding 2-week period. Compliance will be assessed and AEs will also be assessed with an open-ended question. Subjects will be reminded of study instructions (for study beverage consumption, daily Study Log completion, and to maintain habitual exercise, meal/diet and medication/supplementation use). Week 10 check in may take place as a video-conference in order to supervise and provide advice on self-application of the continuous glucose and ketone sensor to the outer upper arm. After Week 10 check in, subjects will be mailed a stool sample collection kit for sample collection up to 3 days ahead of Visit 5.

### Daily at-home procedures (Day 15 - 84)

Each day at home, subjects should consume their first meal of the day at a similar time and consume the study product within 5 minutes of finishing their first meal. Subjects will also complete the Study Log to confirm they consumed their daily study product, and note any symptoms. From Day 15 onwards, a full BTQ is not completed daily. There is space in the Study Log to note any symptoms, these notes will be used to assess tolerance by interview for the previous 2-week interval; at Visit 4, during phone check-ins (Week 6, 8, and 10) and at Visit 5. Study product for the third month will be shipped to arrive in week 7.

### Visit 4: Interim Visit (Week 4 – between Days 28 - 35)

Subjects must meet pre-test requirements on arrival for Visit 4 (fasting ≥ 10 h, no alcohol ≥ 10 h, no exercise ≥ 10 h, no cannabis products ≥ 10 h); a reminder email will be sent out ahead of the visit. On arrival, changes to medication/supplement use will be assessed, and the subject's compliance with inclusion/exclusion criteria will be reviewed. Subjects will be queried to ensure compliance with study instructions. Subjects will be asked to provide any unused product to assess compliance of Study Product consumption, and the Study Log (Part I) will be reviewed and collected. Adherence will be assessed and adverse events (AEs) will also be assessed with an open-ended question. After this a physical assessment will be conducted, including vital signs, body weight and waist circumference.

Fasting blood samples will be collected for the following analysis: clinical chemistry, lipid panel, hematology, liver function and thyroid function. Additional samples will be retained to bank for possible future mechanistic investigations. Then, subjects will be asked to collect a clean catch urine sample (for standard urinalysis). Subjects will verbally complete the BTQ for symptoms occurring in the preceding 2-week period.

Subjects will be reminded of study instructions (for study beverage consumption, daily Study Log completion, and to maintain habitual exercise, meal/diet and medication/supplementation use). Subjects will be given a stool sample collection kit (instructions insert shown in Appendix 3) to take home for at-home sample collection up to 3 days after Visit 4, the stool sample will be returned to investigators using a prepaid mailer. Finally, subjects will be given a 4 weeks supply of study beverages to take home and Study Log (Part II). On this day, subjects will be asked to consume study products with their next meal after the test visit is complete.

The study medical officer will review their safety/tolerability report along with vital signs and the laboratory results within one week, in order to confirm the safety of continued participation in the study.

### Visit 5: Final Visit (Week 12 – between Days 84 - 91)

Subjects must meet pre-test requirements on arrival for Visit 5 (fasting ≥ 10 h, no alcohol ≥ 10 h, no exercise ≥ 10 h, no cannabis products ≥ 10 h). On arrival subjects will be queried to ensure compliance with study instructions, changes to medication/supplement use will be assessed, and subject’s compliance with inclusion/exclusion criteria will be reviewed. Subjects will be asked to provide any unused product to assess compliance of Study Product consumption, and the Study Log (Part II) will be reviewed and collected. After this a physical assessment will be conducted, including vital signs, body weight and waist circumference. If worn, the continuous glucose and ketone sensor will be removed from the outer upper arm.

Fasting blood samples will be collected for the following analysis: clinical chemistry, hematology, lipid panel, liver function, thyroid function and aging biomarker investigations. Additional samples will be retained to bank for possible future additional mechanistic aging biomarker investigations. Then, subjects will be asked to collect a clean catch urine sample (for urinalysis). Subjects will be given access to a selection of snacks after these samples are collected.

Then, a study team member will conduct an interview to assess Katz’s Activities of Daily Living (ADL)^27^and Lawton’s Instrumental Activities of Daily Living (IADLs)^28^and CSHA Frailty Score (Canadian Study of Health and Aging).^29^ Subjects will complete the following paper questionnaires: Profile of Mood States (Short form),^30^The Sexual Quality of Life Questionnaire (Male or Female Version),^31, 32^ Short Form Health Survey-36,^33^Pittsburgh Sleep Quality Index,^36^ Pittsburgh Fatiguability Scale,^34^ Geriatric Depression Scale.^35^(Appendix 4). Subjects will answer the question “do you think you have been consuming ketone or placebo products during the study” (in order to assess the success of blinding) and they will complete an “End of Study Feedback Questionnaire.” After these questionnaires are complete, body weight, waist circumference and vital signs will be recorded.

Subjects will complete cognitive function testing: Montreal Cognitive Assessment,^36^Digit Symbol Substitution Task^37^ and Trails A and B^38^ (Appendix 4) Subjects will complete physical function testing: Short Physical Performance Battery,^39^ 1 rep max leg press,^40^ sub maximal leg press repetitions to failure, 6-minute walk test ^41^and grip strength.^42^

### 7.2.11 Visit 6: Optional Acute Kinetics Visit (Post Week 12)

Subjects who have already completed the 12-week study period will be contacted to ask if they are willing to participate in this optional acute kinetics visit. They will be sent information about the visit ahead of their scheduled appointment, and asked to arrive meeting the pre-test criteria (below). On arrival study staff will explain the rationale for the additional visit, and remind subjects of the kinetics testing procedures. Subjects can ask any questions, and then will sign an updated consent form before any procedures take place. Subjects must meet pre-test requirements on arrival for Visit 6 (fasting ≥10h, no alcohol ≥10h, no exercise ≥10h, no cannabis products ≥10h); a reminder email will be sent out ahead of the visit. Subjects will be queried on any changes in their health that may affect their inclusion in the study. This visit will follow identical procedures to Visit 2 (see above) EXCEPT the KE product form will be a powder reconstituted with water (8 oz or 16 oz for 12.5 and 25 g KE respectively). Subjects will consume the same amount of KE as Visit 2.

## Study Schedule Summary

| **Visit Name** | **Phone Screen** | **Screening/ Enrollment Visit (1)** | **Kinetics Visit**  **Visit (2)** | **Baseline Visit (3)** | **At Home** | **At Home** | **Interim Visit (4)** | **Phone Check In** | **Final Visit (5)** | **Optional**  **Kinetics**  **Visit (6)** |
| --- | --- | --- | --- | --- | --- | --- | --- | --- | --- | --- |
| **Refers to section** | **7.2.2** | **7.2.3** | **7.2.4** | **7.2.5** | **7.2.6** | **7.2.7** | **7.2.8** | **7.2.9** | **7.2.10** | **7.2.11** |
| **Time** |  | **Day -56 to -21** | **Day -42 to -7** | **Day 0** | **Days 1 to 14** | **Days 15 to 84** | **Week 4** | **Weeks 2, 6, 8 and 10** | **Week 12** | **1-20 weeks Post Visit 5** |
| Demographics | X | X |  |  |  |  |  |  |  |  |
| Inclusion/Exclusion | X | X | X | X |  |  | X | X | X | X |
| Interview for Frailty Measures ^1^ | X | X |  |  |  |  |  |  | X |  |
| Informed Consent |  | X |  |  |  |  |  |  |  |  |
| Clinic Visit ^2^ |  | X | X | X |  |  | X |  | X | X |
| Review Medical History & Medication History |  | X |  | X |  |  | X | X | X |  |
| Diet Recall ^3^ |  |  |  | X |  |  |  |  | X |  |
| Vitals/Anthropometrics ^4^ |  | X |  | X |  |  | X |  | X |  |
| Venous Blood Sample and Urine Collection ^5^ |  | X |  | X |  |  | X |  | X |  |
| Study Beverage Palatability Assessment |  | X |  |  |  |  |  |  |  |  |
| Review Study Instructions and Compliance |  | X |  | X |  |  | X | X | X |  |
| Randomization |  | X |  |  |  |  |  |  |  |  |
| Adherence monitoring^6^ |  |  |  |  |  |  | X | X | X |  |
| Physical Testing |  |  |  | X |  |  |  |  | X |  |
| Cognitive Testing^8^ |  |  |  | X |  |  |  |  | X |  |
| Study Product Consumption^9^ |  |  | X |  | X | X |  |  |  | X |
| Capillary Blood Sampling^10^ |  |  | X |  |  |  |  |  |  | X |
| Study Questionnaires^11^ |  |  |  | X |  |  |  |  | X |  |
| Assess tolerability^12^ |  |  | X |  | X | X | X | X | X | X |
| Stool sample collection^13^ |  |  | X | X | X |  | X |  | X |  |
| Continuous glucose and ketone sensor^14^ |  |  |  | X | X |  |  | X | X |  |
| Dispense wrist actigraphy device |  |  | X |  |  |  |  |  |  |  |

**Footnotes**:

^1^ Katz index assesses ADLs and IADLs. Modified questions will be used during the phone screen. Full assessment will occur at Screening and Week 12.

^2^ At least 14 days must elapse between Visit 1 and Visit 2. Visit 1 must occur within 8 weeks (64 days) of Visit 3. At least 7 days must elapse between Visit 2 and Visit 3. Visit 4 must occur no earlier than 28 days and no later than 35 days after Visit 3. Visit 5 must occur no earlier than 84 days and no later than 91 days after Visit 3. At all clinic visits, subjects will be required to arrive in a fasting state (≥10 h), having avoided exercise, alcohol and cannabis products for ≥ 10 h and queried about compliance with this instruction.

^3^ Subjects will complete a 24h diet recall (using the online tool- ASA24) for one day during the week prior to baseline visit and for one day during the week prior to the final visit.

^4^ Body weight, height (Visit 1 only), waist circumference, heart rate, standing and seated blood pressure. At Visit 1, BMI will be calculated

^5^ Fasting (≥ 10 h) blood samples will be collected for the following analysis: hematology, lipids, liver function, thyroid function, clinical chemistry and aging biomarkers (Visit 3 and 5 only). If a clinically relevant laboratory finding occurs at any time point, subject will be removed from the study. Additional sample will be processed to aliquots which will be archived for possible future analyses of mechanistic biomarkers of aging.

^6^ Adherence monitoring will involve review of the Study Log (in person and during phone check in: Weeks 6, 8 and 10) and collection of all unused product bottles at in person visits. Subjects will be instructed to maintain habitual diet, meal schedule, physical activity, and medication and supplement use throughout the study.

^7^ Short Physical Performance Battery ^26^, 1 rep max leg press, submaximal leg press repetition to failure, 6-minute walk test and grip strength

^8^ Montreal Cognitive Assessment, Digit Symbol Substitution Task and Trails A & B.

^9^ During Kinetics visits (2 and 6) subjects will consume a provided breakfast meal followed by Study Product containing 12.5 g of KE, or 25 g of KE. They will consume half a bottle of product containing 12.5 g of KE or canola oil daily at home on Days 1 – 7 after the first meal of the day. On Day 8 at home after their first meal of the day they will consume a full bottle of product containing 25 g of KE or canola oil daily at home; with identical Study Products being consumed daily for the remainder of the Study.

^10^ On Visits 2 and 6 a capillary blood sample will be obtained from a fingerstick prior to Study Product consumption, for real-time analysis of BHB and glucose using a handheld analyzer. Then, subjects will consume a provided breakfast followed by the Study Product. Additional capillary blood samples will be obtained 30, 60, 90, 120, 180 and 240 minutes after Study Product consumption.

^11^ Subjects will complete the following paper questionnaires: Subjects will complete the following paper questionnaires: Profile of Mood States (POMS – short form),^34^ The Sexual Quality of Life Questionnaire (Male or Female Version), Short Form Health Survey-36, ^35^ Pittsburgh Fatiguability Scale, Pittsburgh Sleep Quality Index,^36^ Geriatric Depression Scale.^37^

^12^ Tolerability assessed using BTQ. BTQ will be completed before and after Study Product consumption on Visit 2. BTQ will be completed daily at home, 3 - 6h after study product consumption for the first 2 weeks of the Study. BTQ will be completed verbally during Visit 4 and Visit 5 and also during bi-weekly phone check in (Weeks 6, 8, 10). Adverse events and serious adverse events will be assessed during in person visits (Visits 4 and 5) and during phone check in.

^13^ Subjects will be given stool sample collections kits (5 over the course of the study) and asked to collect a sample up to 3 days before Visit 2 (sample 1), up to 3 days before Visit 3 (sample 2), between 6-9 days after Visit 3 (sample 3), up to 3 days after Visit 4 (sample 4) and up to 3 days before Visit 5 (sample 5). Samples will be returned to investigators using prepaid mailers.

^14^ If available, the continuous glucose and ketone sensor will be self-applied to the outer upper arm at Visit 3, supervised by the Study Team. It will be worn for 14 days and can be removed and disposed of at home. A second sensor will be applied during Week 10 – the subject can self-apply the sensor during the Week 10 phone check in (which may take place via videoconference if the subject requests additional instruction). The sensor will be removed at the final visit (Day 84).

## Procedures by Clinic Visit

Procedures listed are not necessarily performed in the order below.

### Screening (Visit 1; at least 8 weeks before Visit 3, between Day -64 to -21)

- - Informed consent
  - Interview assessment
    - Medical history
    - Assess prior and current medication/supplement use
    - Frailty assessment – ADL and IADL, CSHA score
    - Review inclusion/exclusion criteria
  - Physical assessment
    - Vital signs (after 5 minutes of sitting)
    - Height
    - Body weight
    - BMI assessment
    - Waist circumference
  - Last menses query, where appropriate
  - Urine sample (clean catch) for urinalysis
  - Fasting chemistry profile
  - Fasting hematology panel
  - Fasting lipid profile
  - Fasting blood thyroid hormones
  - Administer study snack
  - Study beverage palatability evaluation
  - Distribute stool sample collection kit
  - Review study instructions
  - Randomization (after review of laboratory test results)

### Kinetics Visit (Visit 2; at least 2 weeks after Visit 1, at least 1 week before Visit 3, between Day -7 to -42)

- Study instructions query
- Baseline BTQ
- Baseline capillary samples for BHB and glucose
- Administer study breakfast
- Administer study product
- Serial capillary BHB and glucose readings
- Post- product BTQ
- Familiarization with physical function testing
- Dispense actigraphy device (Fitbit)
- Distribute stool sample collection kit
- Online diet recall instructions
- Study instructions reminder

### Baseline (Visit 3; Day 0)

- - Interview assessment
    - Assess concomitant medication/supplement use
    - Review inclusion/exclusion criteria
    - Study instructions query
  - Administer quality of life questionnaires
  - Physical assessment
    - Body weight
    - Waist circumference
    - Vital signs (after 5 minutes of sitting)
  - Sample collection
    - Urine sample (clean catch) for urinalysis collected before beverage consumption
    - Fasting chemistry profile
    - Fasting hematology panel
    - Fasting lipid profile
    - Fasting blood thyroid hormones
    - Aging biomarkers
    - Archive blood samples
  - Administer study snack
  - Conduct cognitive testing: MoCA, DSST, Trails A and B.
  - Conduct physical testing: SPBB, 1 rep max leg press, submaximal leg press to failure, 6-minute walk test, grip strength
  - Distribute Study Log Part I
  - Apply continuous glucose and ketone sensor (if available)
  - Distribute stool sample collection kit
  - Provide study beverages to take-home – 35 days’ supply
  - Study instructions reminder

### Interim Safety Visit (Visit 4; between Day 28 - 35)

- - Interview assessment
    - - Study instructions query
      - Assess concomitant medication/supplement use
      - Review inclusion/exclusion criteria
      - Collect unused study beverage/review log/assess compliance
      - Administer BTQ for period Day 14 - 28.
      - Assess AEs
  - Physical assessment
    - Body weight
    - Waist circumference
    - Vital signs (after 5 minutes of sitting)
  - Sample collection
    - Urine sample (clean catch) for urinalysis collected before beverage consumption
    - Fasting chemistry profile
    - Fasting hematology panel
    - Fasting lipid profile
    - Fasting blood thyroid hormones
    - Archive blood samples
  - Administer study snack
  - Collect Study Log Part I
  - Distribute Study Log Part II
  - Provide a 35 day supply of study beverage to bring home
  - Distribute stool sample collection kit
  - Study instructions reminder

### Phone Check In (Weeks 2,6, 8 and 10)

- - Assess concomitant medication/supplement use
  - Review inclusion/exclusion criteria
  - Assess AEs
  - Review log/assess compliance
  - Administer BTQ for the period 14 days prior to call
  - Study instructions reminder
  - Week 10 only: supervise placement of continuous glucose and ketone sensor (if available).

### Final Study Visit (Visit 5; between Day 84 – 91)

- - Interview assessment
    - Study instructions query
    - Assess concomitant medication/supplement use
    - Review inclusion/exclusion criteria
    - Frailty assessment – ADL and IADL, CSHA score
    - Collect unused study beverage/review log/assess compliance
    - Administer BTQ for period Day 70 - 84
    - Assess AEs
  - Administer quality of life questionnaires
  - Physical assessment
    - Vital signs
    - Body weight
    - Waist circumference
  - Sample collection
    - Urine sample (clean catch) for urinalysis collected before beverage consumption
    - Fasting chemistry profile
    - Fasting hematology panel
    - Fasting lipid profile
    - Fasting blood thyroid hormones
    - Aging biomarkers
    - Archive blood samples
  - Administer study snack
  - Conduct cognitive testing: MoCA, DSST, Trails A and B.
  - Conduct physical testing: SPBB, 1 rep max leg press, sub maximal leg press to failure, 6-minute walk test, grip strength
  - Remove continuous glucose and ketone sensor (if used)
  - Blinding assessment – subject asked what study product they think they had been consuming.
  - Collect Study Log Part II

### Optional Kinetics Visit (Visit 6; 1 – 20 weeks Post Visit 5)

- Re-consent subjects who completed the 12- week protocol
- Health conditions update
- Study instructions query
- Baseline BTQ
- Baseline capillary samples for BHB and glucose
- Administer study breakfast
- Administer study product (matched amount to Visit 2)
- Serial capillary BHB and glucose readings
- Post- product BTQ

## Study Beverages

### Description

- All study products are provided as 75 mL bottles of tropical flavored beverage.
- Ketone ester: 75 mL tropical flavored beverage containing 25 g of ketone ester.
  - During kinetics testing (Visit 2), subjects will consume EITHER 12.5 g OR 25 g of ketone ester – serving size randomly allocated.
  - During 12-week study, half a bottle (12.5 g/day) will be consumed for the first 7 days and a complete bottle (25 g/day) will be consumed for the remaining 77 days.
- Placebo: 75 mL beverage containing 25 g non-ketogenic canola oil.
  - During 12-week study, half a bottle (12.5 g/day) will be consumed for the first 7 days and a complete bottle (25 g/day) will be consumed for the remaining 77 days.

1 – 20 weeks post Visit 5 there will be in option for the Study Subjects to take part in a second Acute Ketone Kinetics visit (Visit 6) where an alternate formulation of the ketone ester in powder format will be taken reconstituted in water (8 or 16 oz for 12.5 or 25 g of KE respectively). The amount of ketone ester given will reflect the serving size consumed on Visit 2.

For 12-week study, subjects will be dispensed study beverages and instructed to consume half of one bottle (Days 1-7) or one full bottle (Days 8 – 84) of the beverage per day, within 5 minutes after consuming their first meal of the day, daily.

The C6 Ketone di-ester has been shown to be safe for human consumption and has self-affirmed Generally Recognized As Safe (GRAS) food ingredient status. Commercial distribution of the C6 ketone di-ester has been underway in the USA since early 2021 with no serious adverse events reported. C8 ketone di-ester is a ketone ester product that has a similar metabolic fate (Figure 1), safety and functional properties as C6 ketone di-ester (See Safety Assessment, Kruger 2022), as well as to other ketone-based FDA notified GRAS substances (GRN 515, GRN 1032). C8 ketone di-ester obtained a GRAS conclusion in August 2023, and commercial products containing C8 ketone di-ester are now available. At the doses given in this study, blood BHB concentration will not exceed 5 mM and so there is no risk of ketoacidosis. There is a small risk that the KE drinks may cause acute, mild or moderate side effects (nausea, headache, dizziness) that typically resolve within 90 minutes of drink consumption.

Canola oil has also been shown to be safe for human consumption and is a common, commercially distributed foodstuff. It possesses a Generally Recognized As Safe (GRAS food ingredient status^43^. There are no known serious adverse effects associated with ingesting canola oil.

### Study Beverage Nutritional Facts

| **Study Beverage** | **Active Ingredient** | **Inactive Ingredients** | **Quantity** | **Nutritional Facts** | **Storage Conditions** | **Potential allergens** |
| --- | --- | --- | --- | --- | --- | --- |
| Active | C8 ketone di-ester | Water, high fat whey protein concentrate, modified gum acacia, citric acid, soy lecithin, natural flavors, stevia leaf extract, pectin, sodium carboxymethyl cellulose, potassium sorbate. | 25 g in 75 mL | 240 kcal, 0.5 g fat, 20 mg sodium, 2 g carbohydrate, 2 g protein | Chilled | Dairy and soy |
| Placebo control | Canola oil | Water, high fat whey protein concentrate, modified gum acacia, citric acid, soy lecithin, natural and artificial flavors, stevia leaf extract, pectin, sodium carboxymethyl cellulose, potassium sorbate. | 25 g in 75 mL | 246 kcal, 25 g fat, 20 mg sodium, 2 g carbohydrate, 2 g protein | Chilled | Dairy and soy |
| Active Ketone powder | C8 ketone di-ester | Soluble corn fiber, sodium caseinate | 25g per 38g powder | 240 kcal, 25g fat, 35 mg sodium, 3g carbohydrate, 2g protein | Room temperature | Dairy |

### Labeling and Packaging

The study beverage will be labeled according to the requirements of ICH-GCP guidelines and applicable local regulatory guidelines. Study beverages will be randomized and coded by study center staff who are not otherwise involved in data collection or analysis. BHB Therapeutics will provide individual bottles (single serving) of placebo and the active study beverage. The study blinding team will be responsible for labeling with the protocol number, beverage code, batch or lot number, manufacturer name, consumption instructions and the statement “For Investigational Use Only.” Overage supply will be kept by study staff for use if additional beverages are needed.

### Storage and Dispensing

The study beverages will be stored in a dry secure location and kept chilled (~4 °C).

Study beverages will be dispensed at Visits 3 and 4 (Days 0 and 28). Subjects will be provided with 35 bottles of study beverage at Visit 3 (Day 0), 35 bottles at Visit 4 (Day 28) and 35 bottles by mail in Week 7. This will provide subjects with enough study beverages for flexibility in scheduling. Subjects are required to return all unused study beverages at the next study visit (Visit 4 and 5, Days 28 and 84). Subjects will be instructed to consume half of one bottle (Days 0 - 7) and one full bottle (Days 8 – 84), within 5 minutes after their first of the day meal, daily.

Ketone ester in powder format will be stored at room temperature.

### Blinding

Study products will be labelled and coded by external personnel and Study Staff will remain blinded to the coding of the treatment groups throughout the 12-week study. Study Staff will not be blinded to the coded treatment allocated to each subject, however as the study products will be in identical packaging and are visually identical, they will not be able to differentiate between coded study products. A set of sealed unblinding envelopes will be provided to the Principal Investigator and Medical Officer for use in an emergency situation where knowledge of the study beverage assignment is essential for the subject’s immediate medical care. As study product is being purchased and labelled in batches in order to maximize expiry, ‘re-labelling’ envelopes will be generated during the first product labelling batch and opened by the Study Blinding Team in order to code subsequent batches. Opened re-labelling envelopes will be destroyed after each labelling batch. If unblinding is needed, the Investigator and study staff will open a sealed unblinding envelope to determine the subject’s study beverage assignment. Blinding will be assessed by asking participants if they thought they were taking a ketone or placebo beverage at the end of the final visit, their answer will be recorded in the CRF.

### Access to Randomization Code

The blinding code of the study beverage must be broken only in exceptional circumstances, such as when knowledge of the study beverage is essential for treating a subject due to a Serious Adverse Event (SAE).

Subjects will be given a Study Information Card with basic study product information, study product code and contact details for the study medical officer and principal investigator, in case they need to share with medical professionals that they are involved in a research study and unblinding becomes important for their care.

**Subject Study information card:**

| Front | Back |
| --- | --- |
| **Participant in Research Study**  Blinded Study Product Code: P2201-TDY  Study product contains milk, soy and may contain  either ketone ester or canola oil (25 g per day).  Ketone ester has been associated with GI upset,  dizziness, light-headedness, nausea, cramping,  diarrhea, mild acidosis, mild hypoglycemia, and  mild hypokalemia. Also may affect liver  transaminases (higher) or blood pressure (lower). | If unblinding to confirm the nature of the study product is important for medical care, please call Study Medical Officers (Dr Michi Yukawa and Dr Jennifer Morris) on: (415) 209-2097  If you are unable to reach them, please call the Principal Investigator (Dr John Newman, MD, PhD) on (415) 209-2072 |
| **Participant in Research Study**  Study Product Code: P2201-OLV  Study product contains milk, soy and may contain  either ketone ester or canola oil (25 g per day).  Ketone ester has been associated with GI upset,  dizziness, light-headedness, nausea, cramping,  diarrhea, mild acidosis, mild hypoglycemia, and  mild hypokalemia. Also may affect liver  transaminases (higher) or blood pressure (lower). | If unblinding to confirm the nature of the study product is important for medical care, please call Study Medical Officers (Dr Michi Yukawa and Dr Jennifer Morris) on: (415) 209-2097  If you are unable to reach them, please call the Principal Investigator (Dr John Newman, MD, PhD) on (415) 209-2072 |

The Medical Officer and Principal Investigator must be contacted immediately if it is necessary to unblind the study beverage randomization for a subject. Whenever possible, these persons should be contacted prior to unblinding. Whenever possible, only the Medical Officer should be unblinded.

Documentation of the unblinding will be made in the source documents indicating the reason for the unblinding and the date and time that unblinding occurred.

## Standard Breakfast – Kinetics Visit

At the kinetics visit (Visit 2), subjects will consume a standard study breakfast before they consume their allocated serving size of ketone ester.

1 serving of Nature's Path Organic Gluten Free Instant Oatmeal


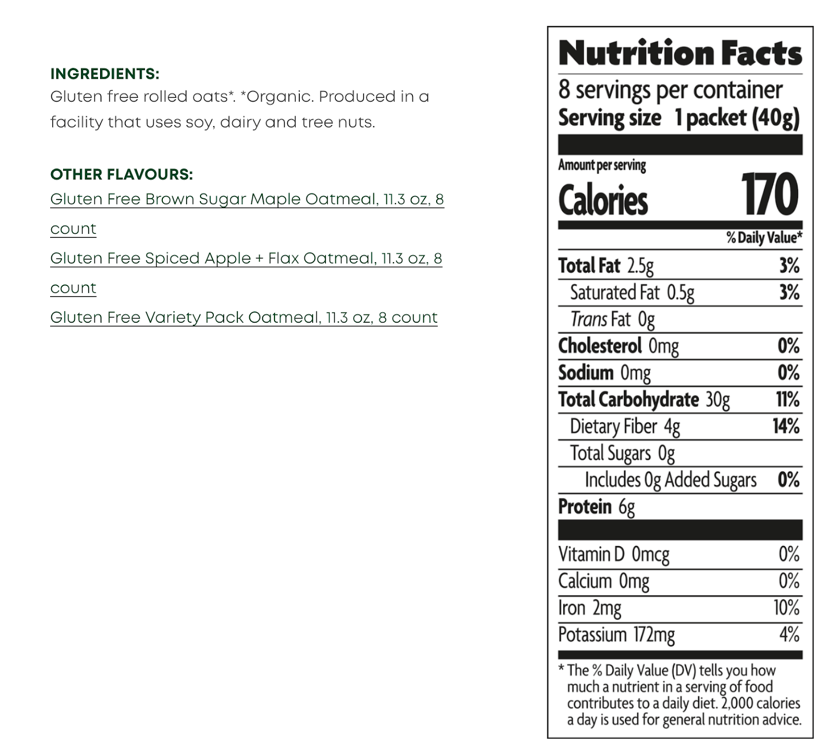


1 scoop of protein powder (Momentous- Vanilla)


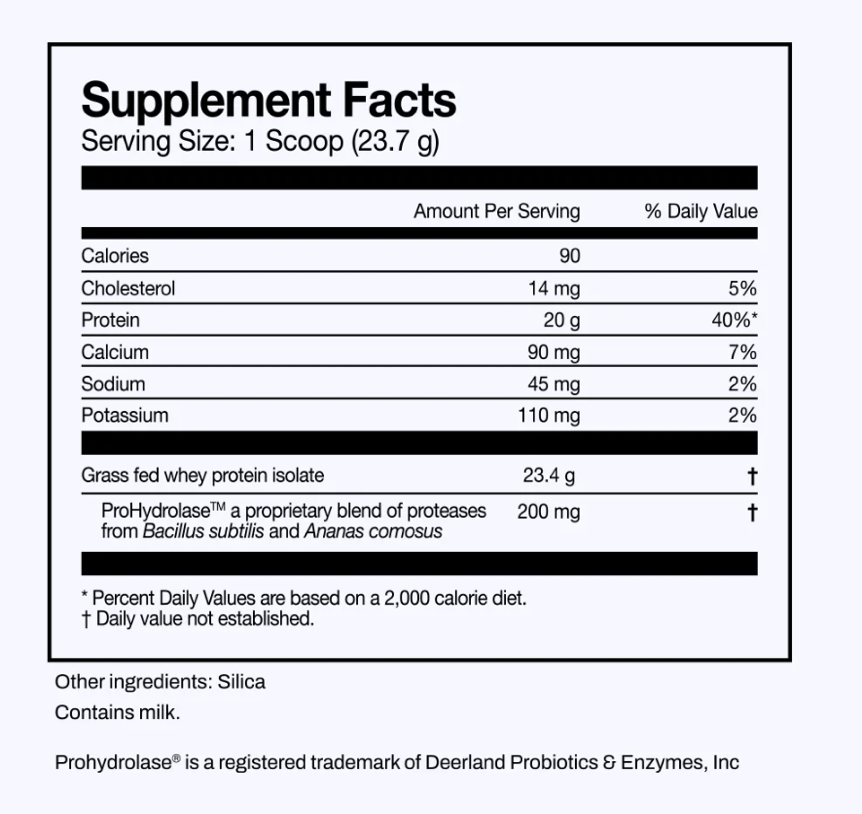


## Clinical Measurements

### Physical characteristics, health updates and vital signs

These include measurement of height (Visit 1 only), body weight, waist circumference, last menses/menopause onset query, and a review of inclusion/exclusion criteria [for eligibility at Visit 1 and for potential protocol deviations at subsequent visits], and concomitant medication/supplement use.

Standardized vital signs measurements will be assessed at each clinic visit and will include heart rate, and both seated and standing blood pressure measured using an automated blood pressure measurement device. Seated blood pressure will be obtained after the subject has been sitting for at least five minutes (after completion of the quality-of-life questionnaires). Standing blood pressure will be measured one minute after subjects’ feet touch the floor using the same arm as the seated measurement. Systolic and diastolic pressures will be measured once using an appropriately sized cuff (bladder within the cuff must encircle ≥ 80% of the arm). Clinic staff may take a repeat measurement, if warranted; the second measurement will be recorded in the CRF.

### Laboratory Measurements

Procedures for all clinical laboratory measurements will be outlined in a laboratory instruction document. Laboratory parameters that are missing or have not been obtained must be entered in the CRF as “not done.” All analytes described below will be assessed by the local Quest Diagnostics Laboratory, unless otherwise indicated.

The following will be performed at Visits 1, 3, 4 and 5 as a part of the fasting (>10 h) blood chemistry profile including, but not limited to, albumin, aspartate aminotransferase, alanine aminotransferase, alkaline phosphatase, total bilirubin, calcium, chloride, creatinine, blood urea nitrogen, potassium, sodium, total protein, carbon dioxide, osmolality and glucose.

The following will be performed at Visits 1, 3, 4 and 5 as a part of the fasting (>10 h) blood hematology including, but not limited to, white blood cell count, red blood cell count, hemoglobin concentration, hematocrit (as volume percent), mean corpuscular volume, mean corpuscular hemoglobin concentration, neutrophils, lymphocytes, monocytes, eosinophils, basophils and platelet count.

The following will be performed at Visits 1, 3, 4 and 5 as a part of the fasting (>10 h) lipid profile including, total cholesterol, triglycerides, HDL, and calculated LDL according to the Friedewald equation.^44^

The following will be performed at Visits 1, 3, 4 and 5 as a part of the fasting (>10 h) thyroid panel: TSH, total T3 and total T4.

A clean-catch urine sample will be collected at Visits 1, 3, 4 and 5 for standard urinalysis. Analytes assessed will include, but not be limited to, glucose, urobilinogen, ketone body, protein, blood cells, bilirubin, specific gravity, and pH.

Blood samples will be collected at Visit 3 and 5 and analyzed at The Buck Institute for aging biomarkers, which may include: DNA methylation of peripheral blood mononuclear cells, cytokine analysis, oxidative stress markers, CRP, sTNFR1, Cystatin C, IGF-1, NT-proBNP, insulin, GDF15, and IGFBPs etc.

Participants will be asked to give consent for cellular and plasma samples to be archived for up to 5 years for post-hoc analyses of aging biomarkers (for example: additional cytokine analysis, DNA methylation analysis), no full genome sequencing will be performed. Samples will be coded with the subject’s study ID, which is only linked to them in a document held by the Investigator in a password protected file on a limited access area on the Buck’s internal secure server. Subjects can withdraw consent for sample storage at any time. Subjects will receive the results from their Quest Laboratory tests before the end of the study. Subjects may request to receive the results of their aging biomarker analysis, these would be shared at the discretion of the PI following completion of analysis and accompanied by a disclaimer that these results are not medically interpretable (Appendix 6).

### Study Instructions/Reminder/Query

Subjects will receive and be reminded of the following study instructions at all visits: maintenance of habitual physical activity, meal schedule, medication/supplement use and diet intake throughout the study, completion of daily Study Log and daily beverage tolerability questionnaires (Days 1-14), and the following prior to study visits: fasting compliance (>10 h, water only), no physical activity, alcohol consumption, or cannabis product use (>10 h). Subjects will be queried about compliance with these instructions at the beginning of Visits 2 - 5.

### Diet Recall

Subjects will complete an online diet recall (online tool: ASA24) for one day in the week before Visit 3 (Day 0) and again during the last week of the study (Day 77 to 84).

Tool: Automated Self-administered 24-hour Dietary Assessment Tool (ASA24^®^)

URL: <https://epi.grants.cancer.gov/asa24/>

Reference: Automated Self-Administered 24-hour (ASA24) Dietary Assessment Tool, version ASA24-2020 (2021), developed by the National Cancer Institute, Bethesda, MD.

Subjects will also be provided the quick start guide found here: [https://epi.grants.cancer.gov/asa24/resourcevs/asa24-quick-start-guide-food-record-03032020.pd](https://epi.grants.cancer.gov/asa24/resourcevs/asa24-quick-start-guide-food-record-03032020.pdf)

Example images:


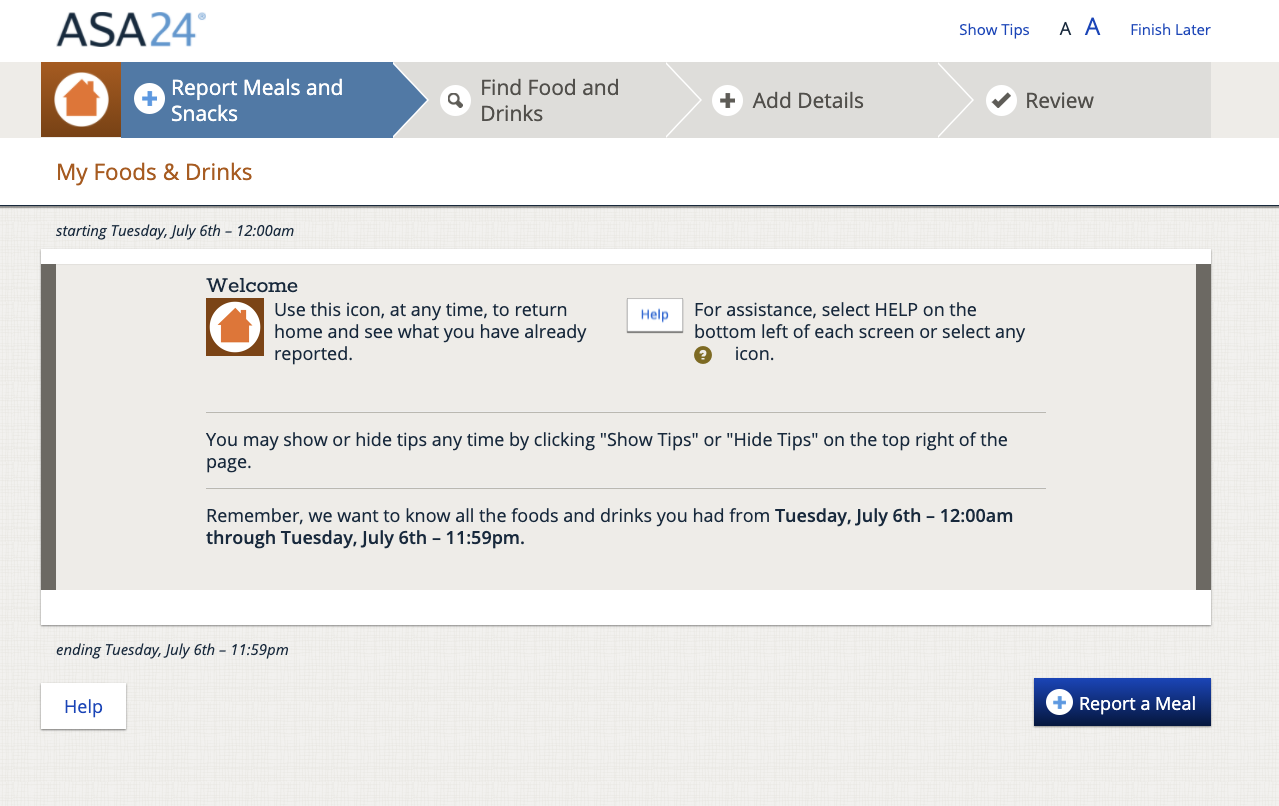

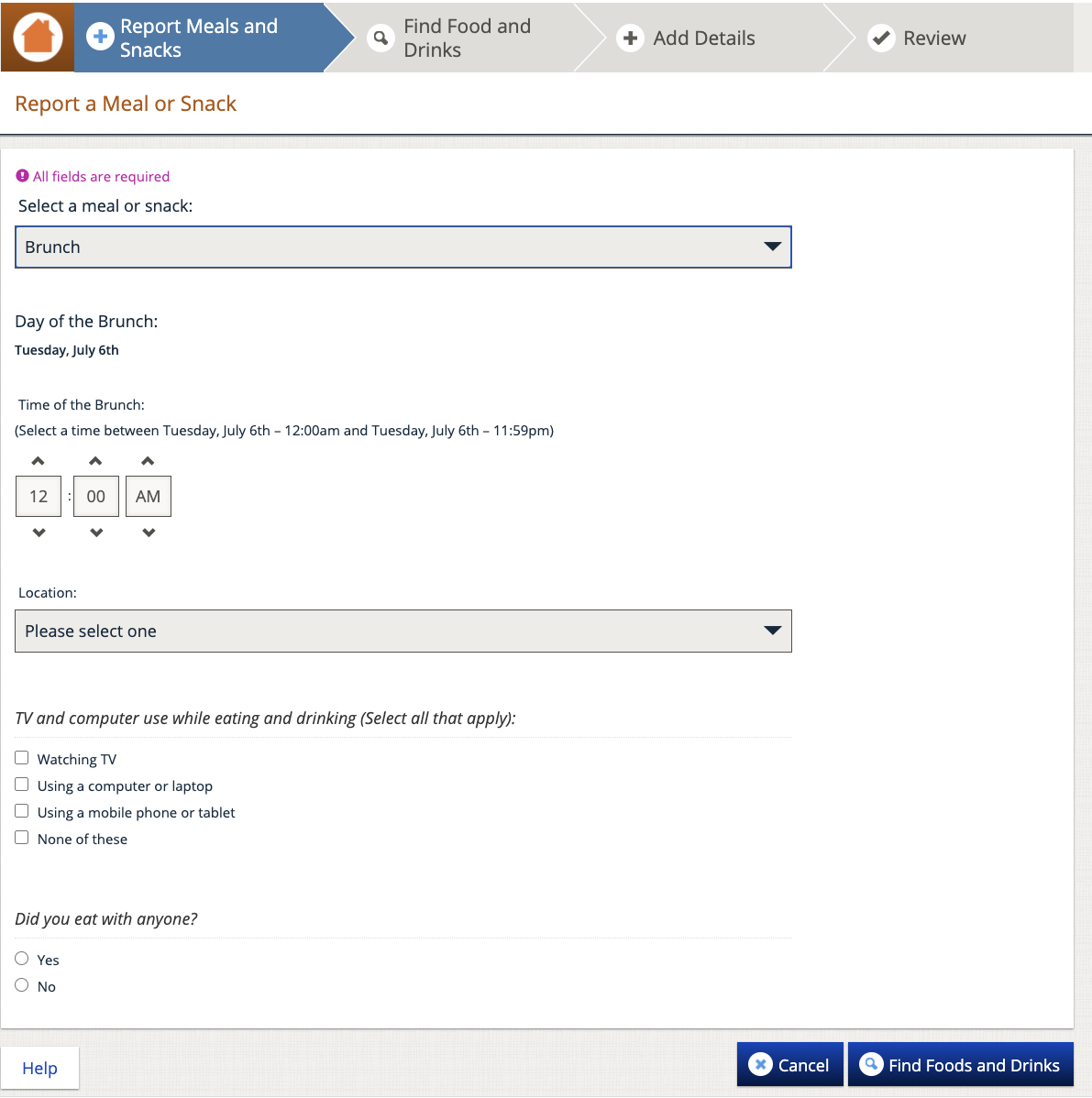

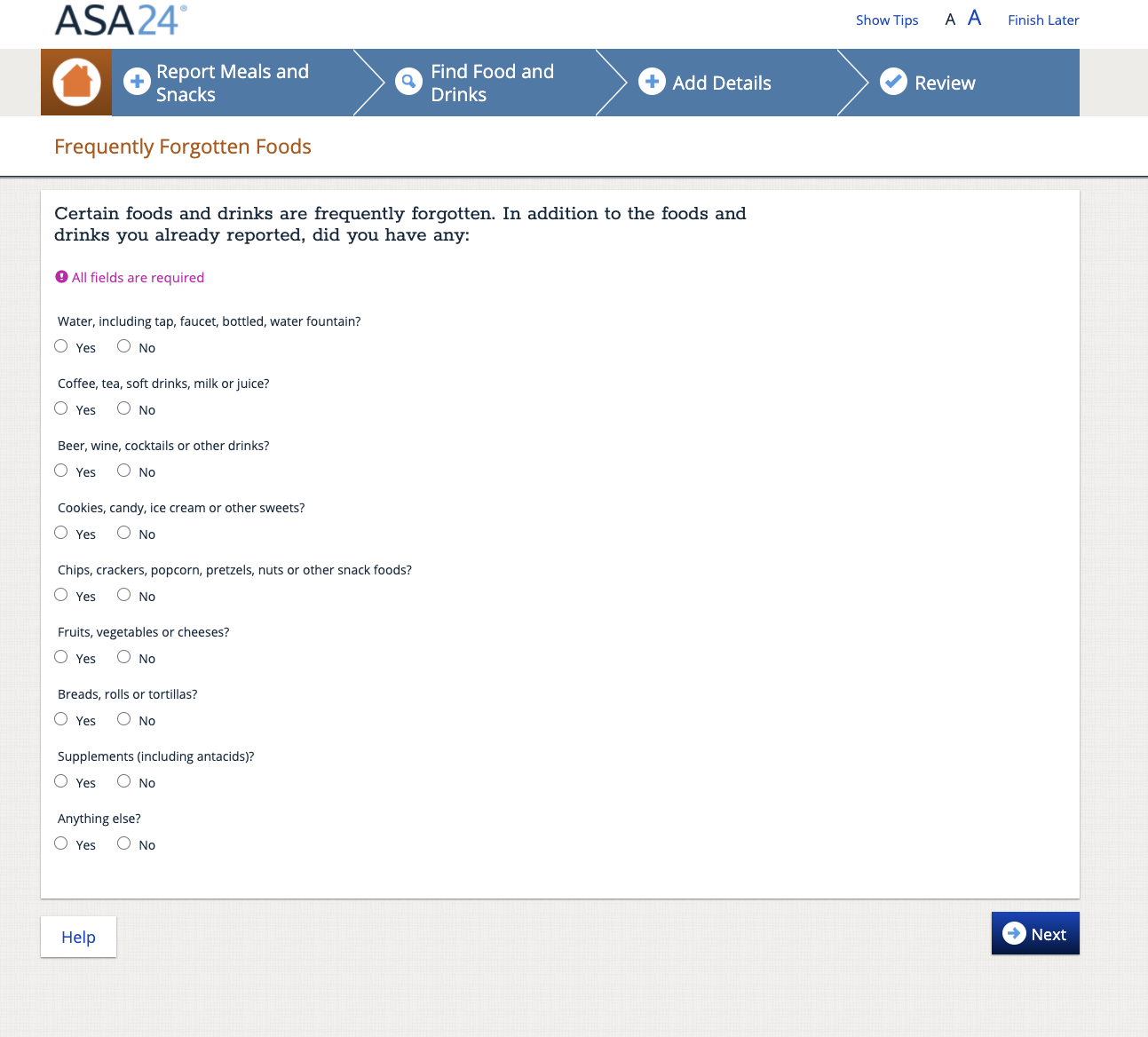


### Study Log and Beverage Tolerability Questionnaire

Study Log (Appendix 5) will allow subjects to check off the study beverages consumed daily throughout the study. Subjects will be asked to return unused study beverages at Visit 4 and 5 (Days 28 and 84). Compliance with the study beverage will be recorded as a percent of scheduled intakes of study beverage consumed based on evaluation of returned unused study beverage and confirmed using the Study Log. Subjects who consume 80-120% of the required study beverage will be regarded as being compliant to the study beverage regimen.

The Study Log will contain space for subjects to complete a Beverage Tolerability Questionnaire (BTQ) once daily at home for the first 14 days, immediately before consumption of the second meal of the day (or 3-6 h post-beverage consumption). The BTQ will include a series of questions regarding the presence and severity of 10 different symptoms including: gas/flatulence, nausea, vomiting, abdominal cramping, stomach rumbling, burping, reflux (heartburn), diarrhea, dizziness and headache ^45, 46^ (see below). Each response is ranked on a 4-point scale ranging from none to severe.

Daily study question: **Since you took the product today, have you experienced any…. (see answers below)**

Tolerance will be queried verbally during the phone check-ins -Weeks 6, 8 and 10.

Phone check in question: **I’d like to ask you to remember any side-effects you might have had after taking the study beverage in the last two weeks. Please review any notes you have in your study beverage log and let me know if you have had any… (see answers below)**

On the Kinetics Visits (Visit 2 and optional Visit 6) subjects will complete a modified version of questionnaire twice: once prior to study breakfast and beverage consumption and the second at 240 min (+/- 10 minutes) post-beverage consumption.

Pre-beverage question: **Please check one answer per line describing if you have any… (see answers below)**

Post-beverage question: **Please check one answer per line describing if you have any… (see answers below)**

**BTQ Answers:**

| **STUDY QUESTION HERE…** | | |
| --- | --- | --- |
| Gas/flatulence? |  | None              Yes |
|  | If yes, is the severity | Mild                Moderate          Severe |
| Nausea? |  | None              Yes |
|  | If yes, is the severity | Mild                Moderate         Severe |
| Vomiting? |  | None              Yes |
|  | If yes, is the severity | Mild                Moderate          Severe |
| Abdominal cramping? |  | None              Yes |
|  | If yes, is the severity | Mild                Moderate          Severe |
| Stomach rumbling? |  | None              Yes |
|  | If yes, is the severity | Mild                Moderate          Severe |
| Burping? |  | None              Yes |
|  | If yes, is the severity | Mild                Moderate         Severe |
| Reflux/heartburn? |  | None              Yes |
|  | If yes, is the severity | Mild                Moderate         Severe |
| Diarrhea? |  | None              Yes |
|  | If yes, is the severity | Mild                Moderate        Severe |
| Headache? |  | None              Yes |
|  | If yes, is the severity | Mild                Moderate         Severe |
| Dizziness? |  | None              Yes |
|  | If yes, is the severity | Mild                Moderate          Severe |

Scoring:

None = 0, Mild = 1, Moderate = 2, Severe = 3.

### Frailty Assessment

During the telephone screen, subjects will be verbally asked a modified version of the ADL questionnaire. At Visits 1 and 5 (Days 0 and 84), eligible subjects will be interviewed by a study team member to assess Katz’s Activities of Daily Living (ADL)^31^ and Lawton’s Instrumental Activities of Daily Living (IADLs)^32^ and CSHA Frailty Score.^33^

### Quality of Life Questionnaires

At Visits 3 and 5 (Days 0 and 84), subjects will complete the following validated questionnaires: Short Form of Profile of Mood States (POMS), ^34^ The Sexual Quality of Life Questionnaire (Male or Female Version), Short Form Health Survey (SF-36),^35^ Pittsburgh Fatiguability Scale, Pittsburgh Sleep Quality Index (PSQI),^36^ Geriatric Depression Scale (GDS).^37^ These assessments are shown in Appendix 4.

### Cognitive Testing

At Visits 3 and 5 (Days 0 and 84), a Study Team Member will administer three standardized cognitive tests that are commonly used in clinical studies: Montreal Cognitive Assessment,^28^ Digit Symbol Substitution Task ^29^ and Trails A and B^30^. These assessments are shown in Appendix 4.

- - - 1. Montreal Cognitive Assessment

The Montreal Cognitive Assessment (MoCA) was designed as a rapid screening instrument for mild cognitive dysfunction. It assesses different cognitive domains: attention and concentration, executive functions, memory, language, visuoconstructional skills, conceptual thinking, calculations, and orientation. There are 11 tasks that will be administered by a trained member of the study team, completed on a certified MoCA testing sheet. The results will be interpreted by a physician. The first is alternate trail making; where subjects must connect . The visualconstructional skills task (#2) requires subjects to firstly copy a shape (e.g., a cube or a bed), and then to draw a clock. The naming task (#3) requires subjects to name three animals. The memory task (#4) requires subjects to remember 5 words and recall them immediately, and also at the end of the test. The attention task (#6) requires subjects to repeat a sequence of numbers in a forward and backward sequence, then subjects must listen to a series of numbers or letters and tap when they hear a pre-specified number/letter, finally subjects must complete a serial subtraction task. Next, subjects must repeat two sentences exactly as given by the investigator (#7). To test verbal fluency, subjects must list as many words as possible starting with a given letter (#8). To test abstraction, subjects must describe the link between two words (i.e., orange and banana) (#9). To test delayed recall subjects must repeat the 5 words from earlier in the test (#10). Finally, to test orientation, subjects must give the date, and the name of the place and city in which the test is being conducted (i.e., The Buck Institute, Novato).

- - - 1. Trails A and B

The trail making test, trails A and trails B, is a simple test that can be used to screen for dementia or cognitive impairment. It consists of two-timed tests that assess a person’s cognitive functions. It does so by determining how fast an individual can search, scan, and process visual information without losing focus. Part A consists of 25 circles on a piece of paper numbered 1 to 25 in each circle. The subject is tasked with drawing a line from one circle to the next in ascending numerical order as quickly as possible. Part A mainly measures working memory. Part B consists of 24 circles on a piece of paper. Half the circles contain numbers, and the other half contain the letters A through L. The subject is tasked with drawing a line from one circle to the next, alternating between numbers and letters in ascending order. Part B mainly measures executive control, including set-shifting and cognitive flexibility. (i.e., 1-A-2-B-3-C-4-D-5-E-6-F-7-G-8-H-9-I-10-J-11-K-12-L).

- - - 1. Digit Symbol Substitution (DSST)

The DSST is a standard neuropsychological test that requires the completion of a timed, pencil and paper task. The DSST requires response speed, sustained attention, visual spatial skills and set shifting. The subject fills a series of symbols correctly coded within 90 seconds. First, the subject practices on a sample, copying the correct symbol given for each number. Then, the subject is timed on the actual task. In this test the higher the score the better the person’s performance.

### Physical Testing

At Visits 3 and 5 (Days 0 and 84), a Study Team Member will administer standardized physical function testing: comprising Short Physical Performance Battery ^26^ [balance testing, 4m gait speed and chair sits], 1 rep maximal leg press and submaximal leg press repetitions to failure, 6-minute walk test and grip strength [measured using a hand dynamometer, duplicate readings taken for each hand]. Standard guidelines will be followed to ensure instructions and measurements are kept consistent between subjects and between visits.

- - - 1. Short Physical Performance Battery

A study team member will guide the subject through the test to ensure the participant is safe from injury. The test has three parts. Firstly, balance testing. Subjects must stand with their feet in three different positions and balance for up to 10 seconds in each position. The three positions are: feet side by side, feet in the semi-tandem position and feet in the tandem position. Time held in each position is recorded in the CRF and scored. Secondly, gait speed testing. Subjects must walk 4 meters at ‘their usual speed’ twice, the time for each repetition is recorded in the CRF and scored. Thirdly, chair stands. Subjects are seated in a chair and stand once (first test) and then five times (second test) without using their arms. The results are recorded in the CRF and scored.

- - - 1. 1-rep maximal leg press (1-RM)

A study team member will guide the subject through the test and ensure good form to minimize the chance of injury. Subjects will sit on the machine and machine settings and foot position will be adjusted to ensure correct knee and hip angles, machine settings and foot position will be recorded in the CRF to maintain consistency between visits. Subjects will complete a warm up set of 5-6 reps with no weight added, and then 4 sets where the weight is gradually increased from 50% expected maximum weight (based on body weight) up to 95% expected maximum (see below). The number of target reps completed in each set decreases from 6-7 to 1-2 as the weight increases. After each set, subjects will rate their exertion using the Borg Scale (below), the RPE should increase with each set. There will be between 1-5 minutes between each set, the time will increase as weight and RPE increases. Set 5 onwards are attempting to find the maximal weight that can be moved for 1 repetition. The 1-RM weight will be recorded in the CRF.

- - - - 1. Target weight, rep number and RPE


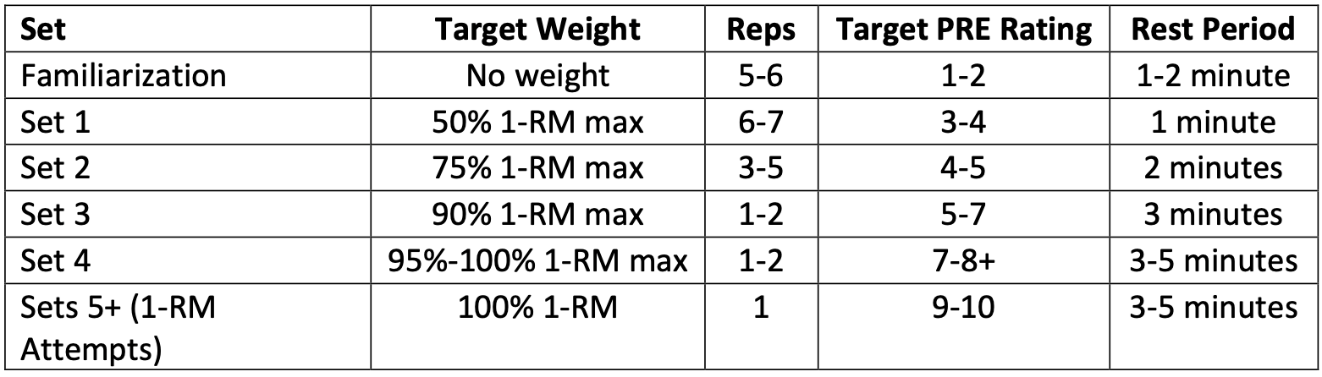


- - - - 1. Borg Scale for Perceived Exertion (RPE)


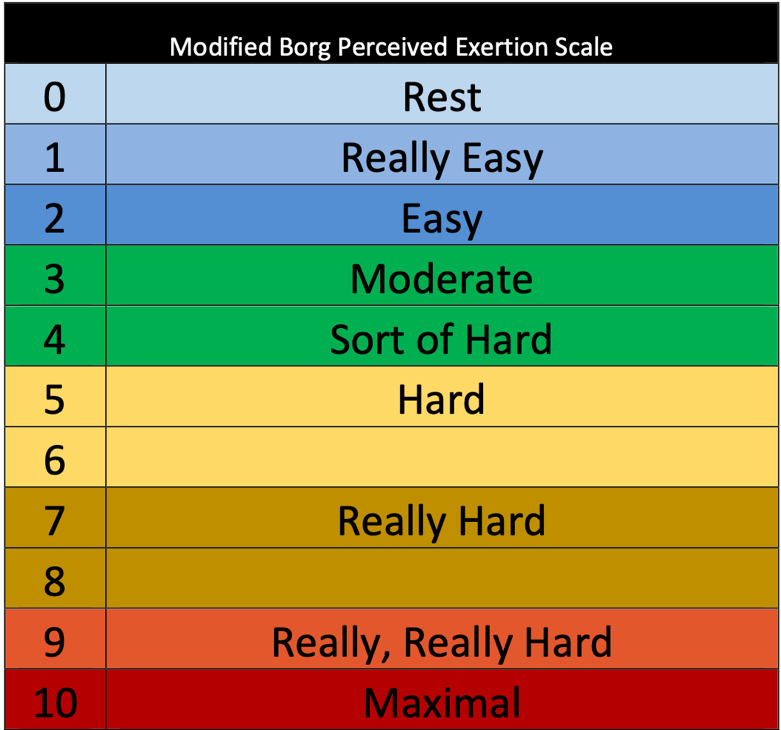


- - - 1. Submaximal leg press to failure

A study team member will guide the subject through the test to ensure the subject is safe from injury. This will take place at least 5 minutes after the 1-rep maximal leg press test . The leg press machine set up and foot position will be unchanged from the earlier test. One warm up set (5-6 reps) with no weight will be completed. A submaximal load (60-70% of 1-RM achieved) will be added to the machine. Subjects will have one attempt to complete as many reps at the submaximal weight as they can, until no further reps can be completed; the weight used and number of reps will be recorded in the CRF. RPE immediately after the exercise will be assessed using the Borg scale and recorded in the CRF.

- - - 1. Grip strength

A study team member will guide the subject through the test to ensure the subject is safe from injury. Subjects will squeeze a hydraulic handheld dynamometer (Jamar) as hard as they can for up to five seconds. The device records the maximum force produced; this will be recorded in the CRF. Subjects will alternate between their right and left hands until three trials per hand have been completed.

- - - 1. 6-minute walk test

A study team member will guide the subject through the test to ensure the subject is safe from injury. A track is marked by cones on a flat surface. The test is explained, and the subject is asked to rate their pre-test RPE using the Borg Scale (above). Subjects then must complete as many laps of the track as they can in 6 minutes. A study team member will count the laps, monitor the time elapsed and provide standard verbal encouragement through the test. The number of laps completed, plus any partial laps, are recorded in the CRF. Subject will record a post-exercise RPE rating.

### Study Beverage/Standard Breakfast Administration

At Kinetics Visits (Visit 2 and optional Visit 6) (Days -7 to – 14 AND at least 1 week after Visit 5) eligible subjects must arrive fasted (>10 h) and having avoided exercise, cannabis products and alcohol for >10 h. Following baseline capillary blood sampling and BTQ subjects will be given a standard breakfast meal immediately followed by their assigned study beverage at t = 0 h. Subjects will consume the study beverage within 5 min.

For the remainder of the study, subjects will be provided study beverages to be consumed at home daily within 5 minutes after their first meal of each day.

At the Optional Acute Ketone Kinetics Visit (Visit 6) (1 – 20 weeks after Visit 5) eligible subjects must arrive fasted (>10 h) and having avoided exercise, cannabis products and alcohol for >10 h. Following baseline capillary blood sampling and BTQ subjects will be given a standard breakfast meal immediately followed by their assigned study beverage at t = 0 h. Subjects will consume the study beverage within 5 min.

### Capillary Testing for Ketones and Glucose

At Kinetics Visit (Visit 2 or Visit 6), subjects will complete a baseline BTQ, and a baseline BHB and glucose reading will be taken using capillary blood obtained from a fingerstick. A lancing device is used, and collected on to glucose/ketone test strips inserted into a clinical grade handheld meter (KetoMojo, Napa, CA). Subjects will be given a standard breakfast meal. Within 5 minutes of finishing the meal, the study beverage will be administered and consumed within 5 minutes; a timer will be started directly after the subject finishes the study beverage. Fingerstick blood samples will be collected for BHB and glucose determination 30, 60, 90, 120, 180 and 240 minutes after study beverage consumption. A second BTQ will be completed 240 minutes after study beverage consumption.

- - 1. Activity Tracking

Participants will be asked to bring a Fitbit compatible device with them to Visit 2. They will be shown how to connect the device to the wearable, how to charge the device and how to ensure the device and wearable are syncing regularly during the study. The subject will be given an account that is set up by and accessible to the study team, who will check data is being uploaded periodically through the study. Examples of outcomes of interest are: moderate to vigorous physical activity (MVPA), sedentary behavior and steps. At the end of the study, subjects can keep the Fitbit device, but they must create a new account that will not be accessible to the study team.

### Continuous Glucose and Ketone sensor

At Visit 3 subjects will be shown how to apply a continuous glucose and ketone sensor to their outer, upper arm. The sensor is under development by Abbott Biowearables and is similar in appearance and function to their widely used Freestyle Libre 3, the world’s smallest and thinnest continuous glucose sensor. The system sends minute by minute glucose and ketone readings to a compatible device, and can be worn for up to 14 days. The sensor can be easily removed by the subject at home on Day 14 of the study and no special disposal is required. A second sensor will be sent to subjects by mail and a short video conference will be arranged to guide them through self-application of the sensor ahead of the final 2 weeks of the study (~ day 70). Use of these sensors is dependent on their continued availability from Abbott, if supply is interrupted and sensors are unavailable, this will be noted in the CRF.

### Stool Sample Collection

Subjects will be provided with stool sample collection kits at Visit 1, 2, 3, 4 and by mail before Visit 5. Kits are supplied with instructions (shown in Appendix 3) and returned using a prepaid mailer. This kit will include collection containers, plastic collection container frames, collection vials, fecal occult blood test (FOBT) cards, thermal envelopes, cold gel packs and disposable gloves. Following defecation into the collection container, the subject will collect two fecal samples using one of two different methods: 1) a FOBT card sample and 2) a scoop sample. For the FOBT card sample, the subject will use a stick, which is provided in the FOBT card test kit, to sample a small portion of stool from the collection container and smear the sample across the area of the FOBT card. The FOBT card will then be folded and placed in a Ziploc bag in a thermal envelope. For the scoop sample, the subject will use the provided scoop, which is attached to bottom insert of the lid accompanying the collection container, to collect a sample of stool (1-2 grams of sample for a full scoop). This sample will be sealed in a plastic Ziploc bag and placed into the thermal envelope along with the freezer pack and mailed via Priority Mail. Collection vials and FOBT cards will be labeled with the subject study ID number: no personal identifiers will be included on the labels. Upon delivery to the laboratory, the samples will be immediately banked and stored at -80°C.

# Data Analysis and Statistical Methods

## Primary Outcome Variables

| **Primary** | The primary outcome measure is the proportion of subjects reporting the same moderate to severe symptom (among dizziness, headache or nausea) occurring on more than one day within any given recall period (after week 0 – 2 acclimation period) when ketone esters are consumed daily for 12 weeks. |
| --- | --- |

## Secondary Outcome Variables

| **Secondary** | Safety: study beverage-emergent adverse events reported by subjects, vital signs (blood pressure, heart rate, body temperature), body weight, and changes in clinical laboratory measurements (chemistry panel [including thyroid hormones], hematology panel, lipid panel [including apolipoprotein B]) when ketone esters are consumed daily for 12 weeks.  Describe the short-term blood ketone and glucose changes in adults ≥65 y after consuming a single serving of either 12.5 g or 25 g of ketone ester. |
| --- | --- |

## Exploratory Outcome Variables

| **Exploratory** | Differences in physical function between groups at the end of the study  Differences in cognitive function between groups at the end of the study  Differences in quality-of-life scores between groups at the end of the study |
| --- | --- |

## Sample Size

Based on studies of symptoms in older adults, ^47, 48^ we predict the primary outcome rate in the placebo group will be approximately 10%. N=30 (15 per arm) provides 36% power to detect a 25% increase (from 10% to 35%) in the proportion of subjects meeting this primary outcome in the KE condition with two-sided α=0.10. If we assume a larger effect of the KE intervention, N = 30 (15 per arm) provides approximately 75% power to detect an increase (from 10% to 55%) in the proportion of subjects meeting this primary outcome in the KE condition, with two-sided α=0.10.

## Statistical Analysis

All analyses are based on intention to treat. The proportion of subjects with the primary outcome will be compared between groups with Fisher's exact test. The frequency of each individual symptom, the frequency of mild symptoms, and the total composite scores will be similarly compared. During the 14-day acclimation period, the daily composite score will be calculated as the sum of the individual items and will be analyzed with a random coefficient model. The within-group change in safety labs will be compared between groups with a Wilcoxon rank sum test and a false discovery rate (FDR) adjustment to control for multiple comparisons. Continuous secondary and exploratory outcomes will be compared between groups with an analysis of covariance (ANCOVA) approach or a repeated measures model. Data from subjects who complete Visit 2 and optional Visit 6 will be compared using paired t-test ort wo way ANOVA with appropriate post hoc corrections for multiple comparisons.

All statistical analyses will be conducted using SAS for Windows (version 9.4, Cary, NC) or GraphPad Prism version 10. The intent-to-treat (ITT) population will comprise data for all subjects who were randomized and consumed one serving of the study beverage. In addition, the per protocol (PP) population will be identified as a subset of the ITT population, in which subjects will be excluded for the following reasons and possibly others:

- Violations of inclusion or exclusion criteria that could influence the evaluation of response
- Non-compliance by the subject, including, but not limited to:
- Use of prohibited drugs or any products thought to alter the primary outcome variable during the study
- less than 80% or more than 120% compliance with study beverage consumption
- Not adhering to instructions as outlined in the protocol

All decisions regarding exclusion from the ITT and PP populations will be documented prior to database lock.

### Outcome Analysis

Analyses will be conducted by an external study statistician, blinded to intervention coding. Descriptive statistics (number of subjects, mean, standard error of the mean (SEM), standard deviation, median, interquartile limits, minimum and maximum) will be presented. Ratings for beverage symptoms will be presented as counts and percentages. All tests of significance, unless otherwise stated, will be performed at alpha = 0.05, two-sided.

Repeated measures analysis of variance (RANOVA) or covariance (RANCOVA) will be used to assess differences between each KE test beverage and placebo for the primary outcome variable. Initial repeated measures RANOVA/RANCOVA models will contain terms for study beverage, time (days 0, 7, 14, 28), study beverage*time interaction, and pre-beverage consumption (where applicable). An adjusted model also considering age and gender will also be included in the analysis. Pairwise comparisons will be performed comparing the placebo to each active study beverage at each time point, and model derived mean estimates (i.e., least squares means (LS-Means) in SAS) will be presented, along with a 95% confidence interval. Model assumptions, such as constant variance and normality of residuals, will be verified using scatter plots and QQ-plots. If indicated, a transformation of the outcome variable may be considered (e.g., rank-based) to compare between study beverage groups.

A generalized linear mixed model may be considered in the event data reflect counts or categorical response.

### Safety Analysis

Safety will be assessed by AEs reported by subjects, as well as assessment of vital signs, body weight, and changes in clinical laboratory assessments.

### Missing or Incomplete Data

Missing data will not be imputed and only observed data will be included in the statistical models.

# Early Termination Procedures

The term “Early Termination” refers to a subject’s non-completion of the study.

Should a subject decide to withdraw, all efforts will be made to complete and report observations as thoroughly as possible. In the event that a subject is withdrawn from the study, the reason for the withdrawal and the party who initiated the withdrawal (subject or Clinical Investigator) will be documented. Should the subject decide to withdraw, documentation of early termination and any AEs and concomitant medication use should be recorded. Study subjects who do not complete the 12 week protocol will not take part in optional Visit 6.

Efforts will be made to retain subjects even if temporary suspension of the study protocol is required due to events below. For example, a mild COVID-19 case may require temporary suspension of the study protocol but the subject will be encouraged to resume the protocol following recovery.

The primary reason for a subject withdrawing prematurely should be selected from the following standard categories:

***Adverse Event*** – event which results in discontinuation of the study beverage by the subject or that in the judgment of the Clinical Investigator for the best interest of the subject requires discontinuation of study beverage (includes all categories of study beverage relatedness; Not Related, Unlikely, Possibly, Probably, and Definitely).

***Death*** – death of the subject.

***Withdrawal of Consent*** – subject desires to withdraw from further participation in the study in the absence of a medical need to withdraw determined by the Clinical Investigator.

***Lost to Follow-Up*** – subject did not return for one or more follow-up visit(s) following dispensing of study beverage and could not be contacted thereafter. The reason for withdrawal was unknown and could not be documented.

***Incident Acute Medical Situation*** – subject experienced an acute medical situation unrelated to the study, that either requires discontinuation of the study beverage or will substantially impact study compliance or outcome measures. In general, this includes any medical situation resulting in hospitalization or an emergency department visit, at the discretion of the investigator.

***Other*** – causes of premature termination from the study other than the above, such as theft or loss of study beverages, termination of study by Sponsor.

# COVID-19 Management Procedures

Additional screening and safety precautions will be undertaken in accordance with CDC and local health authority guidance and Buck Institute Policies in place at the time of screening and study visits. Possible procedures that may be implemented are as follows:

## Recruitment:

- The pre-visit telephone screen may query potential subjects on their COVID-19 risk, which will include diagnosis of COVID-19, presence of COVID-19 related symptoms, and exposure to individuals with confirmed COVID-19.  Those recording relevant risk factors may not be scheduled for a screening visit.

## In-Clinic Study Visits:

- Upon arrival at the clinic site, subjects may be required to complete an online questionnaire querying their COVID-19 risk, which will include diagnosis of COVID-19, presence of COVID-19 related symptoms, and exposure to individuals with confirmed COVID-19. Those recording relevant risk factors could be refused admittance to the site and asked to reschedule their appointment.
- Upon arrival, subjects will be asked to share their COVID-19 vaccination status. As on-site policies change, vaccination status could influence the requirement to take a COVID19 test before entry and/or to wear a mask while on-site.
- Study personnel are required to maintain “up to date” COVID-19 vaccination status per CDC
- In-person visit procedures may be modified to limit contact as much as possible.
- Subject requirements could be implemented, including, but not limited to:
  - Subjects may have gloves, high-quality masks, hand washing stations, tissues, and sanitizer available to them
  - Subjects may be requested to cough and sneeze into their elbow or a tissue and immediately wash hands after and sanitize
  - Subjects may be requested to maintain proper social distancing as much as possible
  - Subjects may be encouraged to avoid touching their face as much as possible
  - Additional safety or screening procedures such as temperature screening could be implemented
- Additional cleaning and infection control measures could be implemented, including, but not limited to:
  - Areas utilized by staff and/or subjects may be sanitized after each use
  - Staff may be required to wear face coverings
  - Staff may be required to maintain social distancing with subjects and other staff

## COVID-19 positive cases:

- If a subject receives a **positive COVID-19** test result but is **asymptomatic** they can remain in the study and may continue to consume study product complete the daily questionnaires at home. They will be asked to miss study visits during the period of CDC-recommended isolation. In general, this is ~5 days after the positive test. They will be encouraged to resume study participation at the end of the isolation period.
- If a subject receives a **positive COVID-19** test result and has **mild symptoms** they can remain in the study, but will pause consumption of study product and completion of daily questionnaires until symptoms are resolved. They will resume the study protocol at the point at which it was suspended, with subsequent study visits delayed accordingly. They will be asked to miss any study visits during the period of CDC-recommended isolation. In general, this is ~5 days after the positive test.
- Subjects who have **severe COVID-19 infection,** defined as requiring emergency department visit or hospitalization, will be withdrawn from the study.

# Study Monitoring

## Concomitant Medication/Supplements and Treatment

All concomitant medications/supplements used 6 months prior to Visit 1 (Day -7 to -14) and during the study will be reported to the study personnel for assessment and recorded in the subject’s CRF.

Use of the medications/supplements described in the “Exclusion Criteria” section is not allowed during this study. If a subject requires any of these medications/supplements, the subject may not enter the study. If a subject begins to use any exclusionary medications/supplements once enrolled, a protocol deviation will be documented.

## Compliance Monitoring

Compliance of study beverage consumption will be recorded as a percent of scheduled intakes of study beverage consumed based on evaluation of returned unused study beverage. The Study Log will be provided to subjects as a paper document. The Study Log will allow subjects to report daily consumption of study beverages consumed and reviewed regularly to ensure subjects remain compliant. Subjects will be asked to return unused study beverages at Visits 4 - 5 (Day 28 and 84) and study beverages will be re-dispensed at Visit 4. Subjects who consume 80-120% of the required study beverage upon study completion will be regarded as being compliant to the study beverage regimen.

## Adverse Event Monitoring

An AE is defined as any untoward medical occurrence in an investigation subject following written informed consent that does not necessarily have a causal relationship with the study beverage. An AE can be any unfavorable or unintended sign (including an abnormal finding), symptom, or disease temporally associated with the use of an investigational product, whether or not related to the investigational product. This includes any occurrence that is new in onset or aggravated in severity or frequency from the baseline condition, or abnormal results of diagnostic procedures (including laboratory test abnormalities). Therefore, clinical observations, including responses to the question, “Have there been any changes in your health or medications since your last visit? For example, have you had to be hospitalized or have you been told you have any new conditions?” will be collected at Visits 2 - 4 (Days 0, 28 and 84). Some side effects or gastrointestinal symptoms could occur as an outcome of this dietary intervention; side effects listed on the BTQ and reported will not be categorized as AEs but recorded as study outcomes. Side effects, outside of what is expected as a result of study beverage consumption, reported by subjects and judged by the Investigators as medically-relevant events and related to study beverage will be recorded as AEs***.***

Events should be considered AEs if they:



- Result in discontinuation from the study,
- Require treatment or any other therapeutic intervention,
- Require further diagnostic evaluation (excluding a repetition of the same procedure to confirm the abnormality),
- Are associated with clinical signs or symptoms judged by the Clinical Investigator to have a significant clinical impact.

### Grading and Severity

The Clinical Investigator will evaluate all AEs with respect to their severity, and record the outcome and action taken on the AE CRF. AEs will be graded as:

**Mild**: Awareness of symptoms but easily tolerated

**Moderate**: Discomfort enough to interfere with but not prevent daily activity

**Severe:** Unable to perform usual activity

### Relationship

The Clinical Investigator will also judge the likelihood that the AE was related to the study beverage and document this on the appropriate CRF as:

| NOT RELATED | This category applies to those adverse experiences which, after careful consideration, are clearly and incontrovertibly due to extraneous causes (disease, environment, etc.). |
| --- | --- |
| UNLIKELY | In general, this category can be considered applicable to those experiences that after careful medical consideration at the time they are evaluated, are judged to be unlikely related to the study beverage. |
| POSSIBLY | This category applies to those adverse experiences for which, after careful medical consideration at the time they are evaluated, a connection with the study beverage administration appears possible and cannot be ruled out with certainty. |
| PROBABLY | This category applies to those adverse experiences that, after careful medical consideration at the time they are evaluated, are felt with a reasonable degree of certainty to be related to the study beverage. |
| DEFINITELY | This category applies to those adverse experiences which the Clinical Investigator feels are clearly related to the study beverage. |

Appropriate therapeutic action and follow-up measures will be performed by the Clinical Investigator in accordance with appropriate medical practice standards of care.

### Serious Adverse Event Definition/Qualification

A SAE is defined as an AE that results in any of the following outcomes:

- Death (note that death is the outcome of a SAE and the cause of death should be listed as the AE),
- Life-threatening event,
- In-patient hospitalization or prolongation of existing hospitalization,
- A persistent or significant disability/incapacity,
- Congenital anomaly or birth defect,
- Any other important medical event that may not result in death, be life-threatening, or require hospitalization, may be considered a SAE when, based upon appropriate medical judgment, the event may jeopardize the subject and may require medical or surgical intervention to prevent one of the outcomes listed above.

In the event of a SAE, the subject may be dropped from the study if the Clinical Investigator deems it necessary.

### Serious Adverse Event Reporting Instructions

If in the opinion of the Clinical Investigator the event meets the criteria of a SAE the following procedures will be followed:

- The Clinical Investigator will report the SAE to the Sponsor and the Medical Officer immediately.
- In addition, the initial SAE report should be submitted with other applicable information (such as medical history, concomitant medications, AEs) to the Sponsor’s representative (Buck Institute Institutional Officer) within 24 h:

Juniper Pennypacker [jpennypacker@buckinstitute.org](mailto:jpennypacker@buckinstitute.org)

Chief of Staff, Vice President of Scientific Operations

- The Clinical Investigator will notify Institutional Review Board (IRB) of the SAE within the parameters and timeframe specified under the IRB Standard Operating Procedures (SOP). An initial report followed promptly by a complete report will be forwarded to the IRB, when applicable.
- Follow-up information relating to a SAE must be submitted to the Site as soon as additional data related to the event are available.
- If a subject is hospitalized or hospitalization is prolonged due to the SAE, the hospital discharge summary will be obtained if possible.
- If a death occurs and an autopsy is performed, a copy of the autopsy report will be obtained if possible.
- All efforts must be undertaken to obtain follow-up information promptly.

### CRF Recording of Adverse Events

All AEs (AE or SAE) will be recorded on the AE CRF page. For subjects who have an ongoing AE at their final study visit, follow-up information will be captured in the AE CRF page which will be completed after 30 days.

### Serious Adverse Event Follow-Up

For all ongoing SAEs occurring during the study, the Clinical Investigator must submit follow-up reports to the Site regarding the subject’s subsequent course. All SAEs that are ongoing at the end of the study or upon discontinuation of the subject’s participation must be followed until either:

- The event resolves, or
- The event/condition has stabilized (e.g*.*, in the case of persistent impairment), or
- The event returns to baseline, if a baseline value is available, or
- The subject dies, or
- The event can be attributed to other than the study beverage, or to other than the study conduct.

### Pregnancy

The study beverages being dispensed for consumption have not been evaluated for safe consumption by pregnant or lactating women and the outcomes measured are expected to be affected by pregnancy and lactation. This study will recruit women over the age of 65, and all women will be confirmed to post-menopausal by self-report. Pregnant or lactating women will be excluded from the study and women of childbearing potential will be required to use appropriate contraceptive methods, which will be documented, to avoid pregnancy.

# Conduct of the Study

## Ethics and Regulatory Considerations

This study will be conducted according to Good Clinical Practice Guidelines, the Declaration of Helsinki (2013),^49^United State Code of Federal Regulation Title 21 and California Protection of Human Subjects in Medical Experimentation Act (sections 24170–24179.5). Signed written informed consent for participation in the study will be obtained from all subjects before protocol-specific procedures are carried out. Subjects will be informed of their right to withdraw from the study at any time.

## Institutional Review Board

The Clinical Investigator will ensure that an appropriately constituted IRB, in compliance with the requirements of 21 CFR 56, reviews and approves the clinical study. Before the study is started, the Clinical Investigator will forward copies of the protocol and consent form for this study to the IRB for review and approval. IRB approval must refer to the study by exact protocol title and number, identify the documents reviewed, and state the date of review. The IRB must be informed of all subsequent protocol amendments. No alterations, modifications to IRB-approved documents, including the protocol, protocol summary, consent form, recruitment materials and questionnaires will be allowed. The IRB must also be informed of all SAEs and of unexpected AEs as outlined in the IRB’s SOPs or reporting guidelines.

## Informed Consent

The study will be explained verbally as well as on the informed consent document. Each subject will be given ample opportunity to inquire about details of the study and to read and understand the consent form before signing it. The language used will be no higher than 8^th^ grade level, and understanding of key study concepts and features will be ensured via teach-back and other interactive techniques. Study personnel who are responsible for obtaining consent will receive training on assessing decision-making capacity and any uncertainties about the decision-making capacity of the subject will result in immediate pausing of the consent process and be immediately escalated to the investigator.

Consent must be documented by the dated signature of the subject. Each subject’s signed informed consent document must be kept on file by the Clinical Investigator for possible inspection by regulatory authorities or by the Sponsor. The subject should receive a copy of the written informed consent document once they have signed it.

A subject may not be admitted to the study unless informed consent of the subject (or their legally authorized representative) has been obtained.

## Subject Confidentiality

The Clinical Investigator is responsible for ensuring that subjects’ anonymity will be maintained. CRFs or other documents will identify subjects by initials, number, or code, and not by name. The Clinical Investigator will keep a separate log showing codes, names, and addresses. All documents showing the subjects’ identity will be kept in strict confidence by the Clinical Investigator. However, the Clinical Investigator agrees that the Sponsor, its employees or agents, the IRB, as well as representatives of the FDA, will have the right to audit and review pertinent medical records relating to this clinical trial and that the subjects will provide written informed consent to this effect.

## Withdrawal of Subjects from the Study

Subjects may be removed from the study for any of the following reasons:

- A subject requests discontinuation;
- The Clinical Investigator initiates removal for medical or compliance reasons;
- Occurrence of any AE or condition that could, in the Clinical Investigator’s opinion, interfere with the evaluation of the effect of the study beverage or put the subject at undue risk.

It is understood by all concerned that an excessive rate of withdrawals can render the study uninterpretable, therefore, unnecessary withdrawal of subjects should be avoided. Should a subject decide to withdraw, all efforts will be made to complete and report observations as thoroughly as possible. In the event that a subject is withdrawn from the study, the reason for the withdrawal will be documented in the CRF.

## Incidental Findings

Several procedures in this study are in clinical use and so could lead to an incidental finding relating to the subject’s medical care. These include: blood test results, blood pressure measurements, Geriatric Depression Scale, Montreal Cognitive Assessment. Any findings of suspected clinical significance will be shared with the Medical Officer for determination of clinical relevance. If relevance is confirmed, subjects will be informed of the finding and advised to share with their personal physician for further assessment and treatment.

## Changes to the Protocol

All changes to the protocol must be documented by amendments to the protocol signed by the Sponsor and the Clinical Investigator. The amended protocol and a revised informed consent form will be submitted for approval to the IRB. A copy of the approval will be provided to the Sponsor. Where the local IRB regulations regarding protocol amendments differ from this policy, the local regulations will apply.

The above-mentioned requirements do not preclude any immediate action from being taken in the interests of subjects' safety.

## Protocol Deviations and Violations

A protocol deviation is a minor departure from the protocol that is approved by the Project Manager prior to implementation and does not compromise subject safety or the integrity of the data. The site should accurately document the deviation and approval in the source document and complete the protocol deviation/violation CRF.

A protocol violation is a divergence from the IRB-approved protocol that is not approved by the Sponsor or authorized designee prior to implementation. A violation can be classified as major or minor. A major violation compromises the safety of the subject or the integrity of the data collected. A minor violation is a less-significant departure from the protocol that, though not pre-approved, does not compromise the safety of the subject or the integrity of the data collected. The site should accurately document the violation in the source document and complete the protocol deviation/violation CRF. Violations that could significantly influence subject safety will be reported to the IRB.

## Case Report Forms

Data collected in the CRF will be documented in an anonymous fashion (e.g*.*, the subject will be identified only by a study number and their initials). Each evaluation recorded in the CRF will be performed at the time specified in the protocol.

All information required by the protocol should be documented in the source records and provided in the CRF. The Clinical Investigator must agree to complete and maintain source documents for each subject participating in the study. An explanation must be given for any omissions. All CRFs must be completed as soon as possible after the subject’s visit, in order that the monitor may verify the validity and completeness of the data. The Clinical Investigator will review and sign (as required) all CRFs for completeness and accuracy. All information on the CRFs must be traceable back to the source documents.

## Monitoring

Remote and on-site monitoring visits will be conducted during the study, focusing on human subject protection and data integrity risks of the trial. A clinical monitoring plan will be developed which identifies specific risk-based monitoring focal points. These may include:

- Informed consent
- Eligibility criteria
- AEs
- Protocol deviations
- Endpoints
- Study beverage accountability

The Site, Clinical Investigator, and any designees will maintain confidentiality of all subject records. During the course of the study, the responsible Site staff will be available to discuss any matters relating to the conduct of the study.

## Auditing

In addition to the monitoring visits outlined above, an investigational site may undergo a quality assurance audit*.* The Sponsor representatives or a regulatory agency may conduct the audit. If a regulatory agency requests an audit of the study site, the Clinical Investigator is required to inform the Sponsor.

## Records and Sample Retention

All study documentation and samples generated in connection with this study will be retained for the greater of five years or time required by applicable federal regulations, and to allow for inspection by the Sponsor’s authorized representatives and/or governmental or regulatory authorities of all such records. Records and samples will be destroyed at the end of the required retention period.

## Termination of Study

The Sponsor and the Clinical Investigator reserve the right to terminate the study at any time. Upon termination, the Sponsor and the Clinical Investigator will assure that adequate consideration is given to protect the interests of each subject.

## Disclosure

By conducting this study, the Clinical Investigator agrees that all information provided will be maintained by the Clinical Investigator and their staff in strict confidence. Such information may be communicated to the Sponsor representative(s) and/or IRB under a similar, appropriate understanding of the confidential nature of the information. Study documents provided (protocols, other material, as necessary) will be stored appropriately to ensure their confidentiality. It is understood that the confidential information provided to the Clinical Investigator will not be disclosed to others without written authorization, except to the extent necessary to obtain informed consent from those subjects who are eligible and choose to participate in the study. Such information will not be provided to potential subjects or subjects by telephone or to any other individual. Conflicts of interest for key study personnel will be reported to the Sponsor.

## References

1. Sato, K., et al., *Insulin, ketone bodies, and mitochondrial energy transduction.* FASEB Journal, 1995. **9**(8): p. 651-8.

2. Newman, J.C. and E. Verdin, *Ketone bodies as signaling metabolites.* Trends in Endocrinology & Metabolism, 2017. **25**(1): p. 42-52.

3. Newman, J.C., et al., *Ketogenic Diet Reduces Midlife Mortality and Improves Memory in Aging Mice.* Cell metabolism, 2017. **26**(3): p. 547-557.e8.

4. Mikkelsen, K.H., et al., *Systemic, cerebral and skeletal muscle ketone body and energy metabolism during acute hyper-D-beta-hydroxybutyratemia in post-absorptive healthy males.* J Clin Endocrinol Metab, 2015. **100**(2): p. 636-43.

5. Cox, P.J., et al., *Nutritional Ketosis Alters Fuel Preference and Thereby Endurance Performance in Athletes.* Cell Metabolism, 2016. **24**: p. 1-13.

6. Evans, M. and B. Egan, *Intermittent Running and Cognitive Performance after Ketone Ester Ingestion.* Med Sci Sports Exerc, 2018. **50**(11): p. 2330-2338.

7. Myette-Côté, É., et al., *A ketone monoester drink reduces the glycemic response to an oral glucose challenge in individuals with obesity: a randomized trial.* The American Journal of Clinical Nutrition, 2019. **110**(6): p. 1491-1501.

8. Neudorf, H., et al., *Oral Ketone Supplementation Acutely Increases Markers of NLRP3 Inflammasome Activation in Human Monocytes.* Molecular Nutrition & Food Research, 2019. **63**(11): p. 1801171.

9. Nielsen, R., et al., *Cardiovascular Effects of Treatment With the Ketone Body 3-Hydroxybutyrate in Chronic Heart Failure Patients.* Circulation, 2019. **139**(18): p. 2129-2141.

10. Kashiwaya, Y., et al., *A ketone ester diet exhibits anxiolytic and cognition-sparing properties, and lessens amyloid and tau pathologies in a mouse model of Alzheimer's disease.* Neurobiol Aging, 2013. **34**(6): p. 1530-9.

11. Ari, C., et al., *Exogenous Ketone supplements reduce anxiety-related behavior in Sprague–Dawley and Wistar Albino Glaxo/Rijswijk Rats.* Front Mol Neurosci, 2016. **9**.

12. Deemer, S.E., et al., *Concentration-Dependent Effects of a Dietary Ketone Ester on Components of Energy Balance in Mice.* Frontiers in Nutrition, 2019. **6**: p. 56.

13. Youm, Y.-H., et al., *The ketone metabolite [beta]-hydroxybutyrate blocks NLRP3 inflammasome-mediated inflammatory disease.* Nat Med, 2015. **21**(3): p. 263-269.

14. Stubbs, B.J., et al., *On the Metabolism of Exogenous Ketones in Humans.* Front Physiol, 2017. **8**: p. 848.

15. Clarke, K., et al., *Kinetics, safety and tolerability of (R)-3-hydroxybutyl (R)-3-hydroxybutyrate in healthy adult subjects.* Regulatory Toxicology and Pharmacology, 2012. **63**(3): p. 401-8.

16. Chen, O., et al., *Tolerability and Safety of a Novel Ketogenic Ester, Bis-Hexanoyl (R)-1,3-Butanediol: A Randomized Controlled Trial in Healthy Adults.* Nutrients, 2021. **13**(6).

17. Leckey, J.J., et al., *Ketone Diester Ingestion Impairs Time-Trial Performance in Professional Cyclists.* Frontiers in Physiology, 2017. **8**: p. 806.

18. Stubbs, B.J., et al., *A randomized, open-label, cross-over pilot study investigating metabolic product kinetics of the palatable novel ketone ester, bis-octanoyl (R)-1,3-butanediol, and bis-hexanoyl (R)-1,3-butanediol ingestion in healthy adults.* Toxicology Research and Application, 2023. **7**.

19. Stubbs, B.J., et al., *In Vitro Stability and In Vivo Pharmacokinetics of the Novel Ketogenic Ester, Bis Hexanoyl (R)-1,3-Butanediol.* Food Chem Toxicol, 2020: p. 111859.

20. Stubbs, B.J., et al., *Toxicological evaluation of the ketogenic ester bis hexanoyl (R)-1,3-butanediol: Subchronic toxicity in Sprague Dawley rats.* Food Chem Toxicol, 2021. **150**: p. 112084.

21. Soto-Mota, A., et al., *Safety and tolerability of sustained exogenous ketosis using ketone monoester drinks for 28 days in healthy adults.* Regul Toxicol Pharmacol, 2019. **109**: p. 104506.

22. Soto‐Mota, A., et al., *Exogenous ketosis in patients with type 2 diabetes: Safety, tolerability and effect on glycaemic control.* Endocrinology, Diabetes & Metabolism, 2021.

23. Evans, M., et al., *No Benefit of Ingestion of a Ketone Monoester Supplement on 10-km Running Performance.* Med Sci Sports Exerc, 2019. **51**(12): p. 2506-2515.

24. Stubbs, B.J., et al., *Gastrointestinal Effects of Exogenous Ketone Drinks are Infrequent, Mild, and Vary According to Ketone Compound and Dose.* Int J Sport Nutr Exerc Metab, 2019. **29**(6): p. 596-603.

25. Vandoorne, T., et al., *Intake of a Ketone Ester Drink during Recovery from Exercise Promotes mTORC1 Signaling but Not Glycogen Resynthesis in Human Muscle.* Front Physiol, 2017. **8**: p. 310.

26. Sengupta, S., et al., *mTORC1 controls fasting-induced ketogenesis and its modulation by ageing.* Nature, 2010. **468**(7327): p. 1100-4.

27. Katz, S., *Assessing self-maintenance: activities of daily living, mobility, and instrumental activities of daily living.* J Am Geriatr Soc, 1983. **31**(12): p. 721-7.

28. Lawton, M.P. and E.M. Brody, *Assessment of Older People: Self-Maintaining and Instrumental Activities of Daily Living.* The Gerontologist, 1969. **9**(3 Part 1): p. 179-186.

29. Rockwood, K., et al., *A global clinical measure of fitness and frailty in elderly people.* CMAJ, 2005. **173**(5): p. 489-95.

30. Curran, S., M. Andrykowski, and J. Studts, *Short Form of the Profile of Mood States (POMS-SF): Psychometric information.. .* Psychological Assessment, 1995. **7**: p. 80-83.

31. Abraham, L., T. Symonds, and M.F. Morris, *Psychometric validation of a sexual quality of life questionnaire for use in men with premature ejaculation or erectile dysfunction.* J Sex Med, 2008. **5**(3): p. 595-601.

32. Symonds, T., M. Boolell, and F. Quirk, *Development of a questionnaire on sexual quality of life in women.* J Sex Marital Ther, 2005. **31**(5): p. 385-97.

33. Walters, S.J. and J.E. Brazier, *What is the relationship between the minimally important difference and health state utility values? The case of the SF-6D.* Health Qual Life Outcomes, 2003. **1**: p. 4.

34. Glynn, N.W., et al., *The Pittsburgh Fatigability scale for older adults: development and validation.* J Am Geriatr Soc, 2015. **63**(1): p. 130-5.

35. Yesavage, J.A. and J.I. Sheikh, *Geriatric Depression Scale (GDS).* Clinical Gerontologist, 2008. **5**(1-2): p. 165-173.

36. Nasreddine, Z.S., et al., *The Montreal Cognitive Assessment, MoCA: a brief screening tool for mild cognitive impairment.* J Am Geriatr Soc, 2005. **53**(4): p. 695-9.

37. Swindell, W.R., et al., *Data mining identifies Digit Symbol Substitution Test score and serum cystatin C as dominant predictors of mortality in older men and women.* Rejuvenation Res, 2012. **15**(4): p. 405-13.

38. Reitan, R.M., *Validity of the Trail Making Test as an Indicator of Organic Brain Damage.* Perceptual and Motor Skills, 2016. **8**(3): p. 271-276.

39. Guralnik, J.M., et al., *A short physical performance battery assessing lower extremity function: association with self-reported disability and prediction of mortality and nursing home admission.* J Gerontol, 1994. **49**(2): p. M85-94.

40. Grgic, J., et al., *Test-Retest Reliability of the One-Repetition Maximum (1RM) Strength Assessment: a Systematic Review.* Sports Med Open, 2020. **6**(1): p. 31.

41. Enright, P.L., *The six-minute walk test.* Respir Care, 2003. **48**(8): p. 783-5.

42. Bohannon, R.W., *Hand-grip dynamometry predicts future outcomes in aging adults.* J Geriatr Phys Ther, 2008. **31**(1): p. 3-10.

43. Dupont, J., et al., *Food safety and health effects of canola oil.* J Am Coll Nutr, 1989. **8**(5): p. 360-75.

44. Friedewald, W.T., R.I. R I Levy, and D.S. Fredrickson, *Estimation of the concentration of low-density lipoprotein cholesterol in plasma, without use of the preparative ultracentrifuge.* Clin Chem, 1972. **18**(6): p. 499-502.

45. Boler, B.M., et al., *Digestive physiological outcomes related to polydextrose and soluble maize fibre consumption by healthy adult men.* Br J Nutr, 2011. **106**(12): p. 1864-71.

46. Maki, K.C., et al., *Fibermalt is well tolerated in healthy men and women at intakes up to 60 g/d: a randomized, double-blind, crossover trial.* Int J Food Sci Nutr, 2013. **64**(3): p. 274-81.

47. Han, M.A., et al., *The impact of medication use on symptom burden in older patients with multiple medical morbidities.* Journal of Clinical Gerontology and Geriatrics, 2013. **4**(3): p. 84-88.

48. Chaudhry, S.I., et al., *Restricting symptoms in the last year of life: a prospective cohort study.* JAMA Intern Med, 2013. **173**(16): p. 1534-40.

49. Assembly, W.M.A.G., *Declaration of Helsinki: ethical principles for medical research involving human subjects.* 2013: JAMA. p. 2191-2194.
