# Supplementary material for: Rationale and protocol for a safety, tolerability and feasibility randomized, parallel arm, double-blind, placebo-controlled, pilot study of a novel ketone ester targeting frailty via immunometabolic geroscience mechanisms
Source: PLoS One. 2024 Sep 18;19(9):e0307951. doi: 10.1371/journal.pone.0307951 (PMC11410252; doi:10.1371/journal.pone.0307951)
Supplement: Attachment — Submitted filename: BIKE Methods Responses FINAL.docx [file pone.0307951.s004.docx]

**Point by point response to changes requested by editors and reviewers:**

**Editors**

1. We have re-checked that the manuscript meets PLOS ONE’s style requirements.
2. We would be happy for the article to be included in the ‘Aging in Human Health and Disease’ collection.
3. We have added the specific patent details in the declarations section of the manuscript: Drs. Newman (US 11,773,051 B2, US 11,608,306 B2) and Stubbs (US 11,645,228 B2) are inventors on patents relating to the use of ketone bodies that are assigned to The Buck Institute. This does not alter our adherence to PLOS ONE policies on sharing data and materials.
4. Ethics statement removed from end of manuscript, leaving it only in methods section.
5. We edited our protocol to remove the confidentiality statement and uploaded a clean copy. We obtained formal written confirmation from the institutional officer that we can comply with this policy, which is uploaded with our resubmission.

**Reviewer 1:**

1. It is unclear why the authors chose the proportion of subjects (binary outcome) reporting the same moderate to severe symptom (among dizziness, headache, or nausea) occurring on more than one day within any given two-week recall period (after week 0–2 acclimation period) to determine the study power instead of the beverage tolerability questionnaire (BTQ) score (continuous outcome).

The primary outcome was decided based on:

1) our prior experience in a similar longer term ketone ester study (PMID: 34208742) and
2) the desire to inform a go-no go decision on further studies with ketone esters in older adults.

In our previous study, we saw the KE was well tolerated in younger adults, with a high proportion of ‘0’ scores on the BTQ, making this not a true continuous variable and requiring modifications to our statistical plan to properly analyze the data. Our statistical team recommended that we use the proportion of subjects as this removed the issue of multiple ‘0’ scores. We selected the specific symptoms of headache, dizziness, and nausea, as they were the most frequently reported symptoms in our young adult study and are potentially problematic in older adults at moderate or severe intensity. The BTQ lacks an established minimal clinically important difference (MCID) in older adults. However, we felt comfortable assigning clinical significance to these specific symptoms at moderate or severe intensity, which permitted us to create a clear, prespecified, go/no-go criteria for clinically meaningful tolerability in this population. As we felt that moderate or severe headache, nausea or dizziness might occur spontaneously in an older adult population, we specified that this must occur on more than one day to trigger the primary outcome. Moderate or severe symptoms were selected because mild symptoms occur at a high rate spontaneously in older adults and moderate/severe symptoms would be the biggest barrier to long-term adherence to an intervention. Thus, our primary outcome is designed to inform if we can move forward with long term KE studies in older adults. This is explained on Page 13 of the manuscript.

2. It is unclear why the authors proposed having an independent study medical officer to review the safety lab results and approve continuation of subjects in the study instead of forming a DSMB.

The purpose of the medical officers was distinct from that of a DSMB. In compliance with the PI’s Conflict of Interest Management plan, the PI was not permitted to be involved in enrollment or continuation decisions. Therefore, the medical officers provided an independent decision on all enrollment and continuation decisions. This was happening in ‘real time’ and was not a periodic review as would be carried out by a DSMB. The IRB did not require use of a DSMB given the overall expected low risk of the study.

3. Please clearly define the “primary outcome” in the Sample Size section. In addition, the authors should focus on a precision analysis instead of a formal power analysis for their pilot study with only 30 participants.

We have added language that clarifies the primary outcome in the sample size section.

We note that in the sample size section (lines 331 – 334), that rather than perform a power calculation to generate a sample size, we discuss the increase in power if there were to be a larger difference between groups given our set sample size of 30 subjects.

4. The authors proposed using block allocation sequence for the randomization process. Please clearly specify the block size.

The block size is now specified in the allocation and blinding section (page 20).

5. In addition to the formal hypothesis test, the authors should focus on the estimation of the study outcomes in the Statistical Analysis section because this is a pilot study.

We have added the following text to the manuscript p22: “The proportion of subjects with at least 2 occurrences of the target symptoms during the post acclimation period will be estimated along with a 90% confidence interval within each group. “

We have expanded on the statement in our initial submission p22: “the frequency of each individual symptom and mild symptoms will be explored with categorical data **methods which includes confidence interval estimation**”

6. Please clearly specify the statistical methods that will be used for generating the 95% confidence intervals for all the study outcomes.

We have added the following information:

“For the primary outcome, the CI will be estimated with the exact binomial confidence interval.”

“Changes in continuous secondary and exploratory outcomes will be compared between groups with an analysis of covariance approach or a repeated measures model, **confidence intervals will be estimated as for the primary outcome or based on the t-distribution as appropriate**”

7. The current length of the paper seems to be too long. The authors may need to move some content to the Supplementary section.

In compliance with the SPIRIT checklist there are a large number elements that must be addressed and at certain points in the manuscript (i.e., the introduction needs to identify the placebo). The length of the manuscript is determined primarily by the requirements of SPIRIT compliance. We are certainly open to recommendations by the editors if certain SPIRIT items should be moved to Supplementary.

**Reviewer 2:**

1. Do not use 'parallel group'

Our study design is to randomly allocate subjects to intervention or treatment groups with no crossover. We believe it is important to be clear that no crossover is involved. We note that this design can be called either ‘parallel group’ or ‘parallel arm’ – so to address this comment we have revised the manuscript to ‘parallel **arm’** throughout.

2. is this pilot?

Yes, this is a pilot study

3. Do not mention pilot

We feel that it is important to highlight that this is a pilot study to provide context on the study goals and the scientific rationale for the sample size and selected outcomes.

4. Headline 'Study design' is okay

5. Overall okay.

We thank the reviewer for their kind words.

**Reviewer 3:**

1. A respectful suggestion: To improve readability and expand readership, the background of the abstract is a dense on lingo that will be foreign to much of the readership (e.g., geroscience interventions, inflammo-metabolic aging mechanisms, geroscience mechanisms). The introduction is outstanding, and simple phrases used there would better set the stage in the abstract. Simpler is better.

Thank you for the feedback – we have simplified the abstract where we can.

2. Many discourage descriptions such as “generalizable” or “representative” to describe clinical trial participants. Often notable differences between those that pursue research study participation and those that do not.

We felt it is important to be clear that the goals of our study would not be met by enrolling an overly (atypically) healthy population, free of chronic disease or medications; or an overly disease-specific population. Either would be wholly unrepresentative of the general population of older adults, as well as unrepresentative of the pre-frail population that is planned for the fully powered follow-up study. We understand the reviewer’s concerns about qualifying exactly what is meant by “representative”, so we changed this to “broad” and added additional explanatory language on page 9.

3. A better description of randomization would be valuable. Are their blocks planned to balance groups on BMI, comorbid conditions, or other parameters? This may ultimately enhance interpretation of data.

Given the small size of this pilot, we believed that additional enrollment block criteria (besides sex) would both comprise randomization and be a barrier to enrollment without adding interpretable data (e.g. there are far more potential comorbid conditions, and perhaps more conditions that have been hypothesized to be impacted by a ketone ester, than planned participants). We added the clarification that ‘no other stratification will occur’ (page 20). We will, however, incorporate the reviewer’s suggestion into plans for a larger follow-up study.

4. The rationale for n = 30 isn’t clear, “Based on studies of symptoms in older adults (43, 44), we predict the primary outcome rate in the placebo group will be approximately 10%. N = 30 (15 per arm) provides 36% power to detect a 25% increase (from 10% to 35%) in the proportion of subjects meeting this primary outcome in the KE condition with two-sided α=0.10.” This would seem to relate to tolerability, so perhaps better to spell out. Moreover, not sure why 36% power is stated favorably. If just a practical sample size, better to state that up front and then provide the power this provides.

We have clarified the description of the sample size selection as follows:

*“N = 30 (15 per arm) was selected as it was a feasible size for this pilot study that provides 36% power..”*

5. Just a comment-- it would be ideal to capture muscle performance and physical function measures on two occasions at baseline to account for learning effects. The placebo arm diminishes this concern, but this is somewhat standard of practice.

Thank you for this feedback. We did consider the tradeoff between additional familiarization versus study burden, cost, and practice effects before settling on the design for this pilot study. The study is now already completed since this paper was submitted so we cannot change the study design now, but we will reconsider this for our planned follow up work.

6. Defer to the editors, but for transparency, it seems the conflicts of Stubbs and Newman should be noted in the main text.

We desire the utmost transparency. Editors – please advise if you would like us to copy the information on our author conflicts from the “Sources of Funding, Author Declarations and Conflict Management” section into the main text or move this section into the main text.

7. Figure 2 is not legible…at all. Fully appreciate this a conversion issue, but it should be reviewed.

We have re-uploaded Figure 2 and will check the resolution in proofs if the article is accepted.
